# Isomalabaricane Triterpenes from the Marine Sponge *Rhabdastrella* sp.

**DOI:** 10.3390/md19040206

**Published:** 2021-04-06

**Authors:** Kuei-Hung Lai, Zheng-Hao Huang, Mohamed El-Shazly, Bo-Rong Peng, Wen-Chi Wei, Jui-Hsin Su

**Affiliations:** 1 PhD Program in Clinical Drug Development of Herbal Medicine, College of Pharmacy, Taipei Medical University, Taipei 11031, Taiwan; 2 Graduate Institute of Pharmacognosy, College of Pharmacy, Taipei Medical University, Taipei 11031, Taiwan; 3 Traditional Herbal Medicine Research Center, Taipei Medical University Hospital, Taipei 11031, Taiwan; 4 National Museum of Marine Biology & Aquarium, Pingtung 94450, Taiwan; zhenghao0503@gmail.com (Z.-H.H.); pengpojung@gmail.com (B.-R.P.); 5 Graduate Institute of Marine Biology, National Dong Hwa University, Pingtung 94450, Taiwan; 6 Department of Pharmaceutical Biology, German University in Cairo, Cairo 11432, Egypt; mohamed.elshazly@pharma.asu.edu.eg; 7 Department of Pharmacognosy and Natural Products Chemistry, Faculty of Pharmacy, Ain-Shams University, Organization of African Unity Street, Abassia, Cairo 11566, Egypt; 8 Division of Basic Chinese Medicine, National Research Institute of Chinese Medicine, Taipei 11221, Taiwan

**Keywords:** isomalabaricane, tetrahydrofuran moiety, anti-leukemia

## Abstract

The marine sponge of the genus *Geodia*, *Jaspis*, *Rhabdastrella*, and *Stelletta* are characterized chemically by a variety of isomalabaricane triterpenes. This class of compounds drew spotlights in marine lead discovery due to their profound anti-proliferative properties. Further research on exploring its chemical diversity led to the identifications of two new isomalabaricane-type triterpenes rhabdastin H (**1**) and rhabdastin I (**2**). Their structures were unraveled using a series of spectroscopic approaches. These isolates were found to exhibit unique structural features with the only reported tetrahydrofuran functionality among all marine-derived isomalabaricanes. Both compounds **1** and **2** showed activities against K562 (IC_50_ 11.7 and 9.8 μM) and Molt4 (IC_50_ 16.5 and 11.0 μM) leukemic cells in MTT cell proliferative assay.

## 1. Introduction

Marine sponges continue to act as a fruitful source of bioactive and unusual metabolites. Among these metabolites are the sponge-derived isomalabaricanes. They were reported to exhibit potent anti-proliferative properties against a series of cancer cells, including colorectal carcinoma [1], ovarian carcinoma [2], acute promyelocytic leukemia [3], acute lymphoblastic leukemia [4], prostate carcinoma [3], gastric adenocarcinoma [3], ductal carcinoma [3], hepatocellular carcinoma [3], cervix carcinoma [3], papillomavirus-related endocervical adenocarcinoma [5], and malignant melanoma [4]. The isomalabaricane-type triterpenoids are characterized by an *α*-methyl group at C-8, owning to their *trans*-*syn*-*trans* tricyclic ring junction instead of *trans*-*anti*-*trans* of their isomers, malabaricanes [1]. Isomalabaricanes are only found in sponges and are considered as chemotaxonomic markers of *Rhabdastrella* sp. [1] Despite the significant difficulties in the isolation and characterization of isomalabaricanes due to light induced 13-*E*/*Z* isomerization [6], their significant cytotoxic activity prompted extensive research on this class of compounds aiming to find new drug leads.

The current study aimed to explore novel isomalabaricanes from marine sources. We isolated two unusual compounds with tetrahydrofuran functional group, the moiety that has been reported previously in malabaricane-type triterpenoids but without any cytotoxic assessments [7,8]. Then, the isolated compounds were evaluated against several cancer cell lines using MTT assay to further interpret their anti-proliferative properties.

## 2. Results

**Figure 1:**
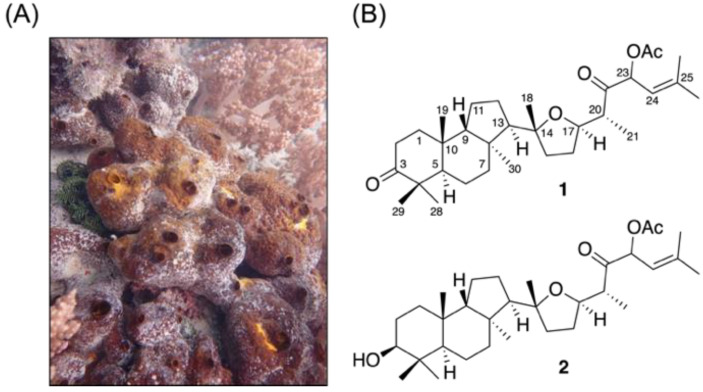
(**A**) Aquatic ecology of the sponge *Rhabdastrella* sp. and (**B**) the isolated isomalabaricane triterpenes.

The freeze-dried specimen of the wild-type sponge *Rhabdastrella* sp. (Figure 1) was extracted with a 1:1 mixture of methanol (MeOH) and dichloromethane (CH_2_Cl_2_) (1:1) to provide the crude extract. The obtained crude extract was further fractionated and purified using normal and reversed-phase column chromatography yielding two isomalabaricanes, rhabdastin H (**1**) and rhabdastin I (**2**). These isolates demonstrated unique structural features with the presence of the first identified tetrahydrofuran moiety in this class of compounds.

The molecular formula of **1** was suggested as C_32_H_50_O_5_ based on ^13^C NMR and HRESIMS data that showed a molecular ion peak at *m*/*z* 537.3539 [M + Na]^+^ implying eight degrees of unsaturation. The IR spectrum of **1** revealed the presence of carbonyl groups from absorptions at 1742, 1726, and 1703 cm^−1^. Its ^13^C NMR spectrum of **1** (Table 1), measured in CDCl_3_, showed the presence of thirty-two carbon signals, which were assigned by the assistance of DEPT data to nine methyl groups, eight sp^3^ methylenes, six sp^3^ methines (including two oxymathines), four sp^3^ quaternary carbons, one sp^2^ methine and four sp^2^ quaternary carbons (including two ketone carbonyl). The NMR signals at *δ*_C_ 169.8 (C) and 20.9 (CH_3_) and *δ*_H_ 2.12 (3H, s) and the IR absorption at 1742 cm^−1^ suggested the presence of an acetoxy group. Carbon signals of the eight methyl groups (*δ*_C_ 31.8, 29.3, 26.0, 24.5, 24.0, 19.3, 19.0 and 12.1), one oxygen-bearing methine carbon (*δ*_C_ 79.8), and one oxygenated quaternary carbon (*δ*_C_ 86.2), one trisubstituted carbon-carbon double bonds (*δ*_C_ 116.9, CH; 142.6, C), two ketones (*δ*_C_ 220.3 and 207.4) were also assigned. The resonances of one olefinic proton (*δ*_H_ 5.11, d, *J* = 10.0 Hz) and two oxygenated methines (*δ*_H_ 6.09, d, *J* = 10.0 Hz; *δ*_H_ 3.76, ddd, *J* = 14.0, 8.5, 4.5 Hz) were observed from the ^1^H NMR spectroscopic data of **1** (Table 1).

**Figure 2:**
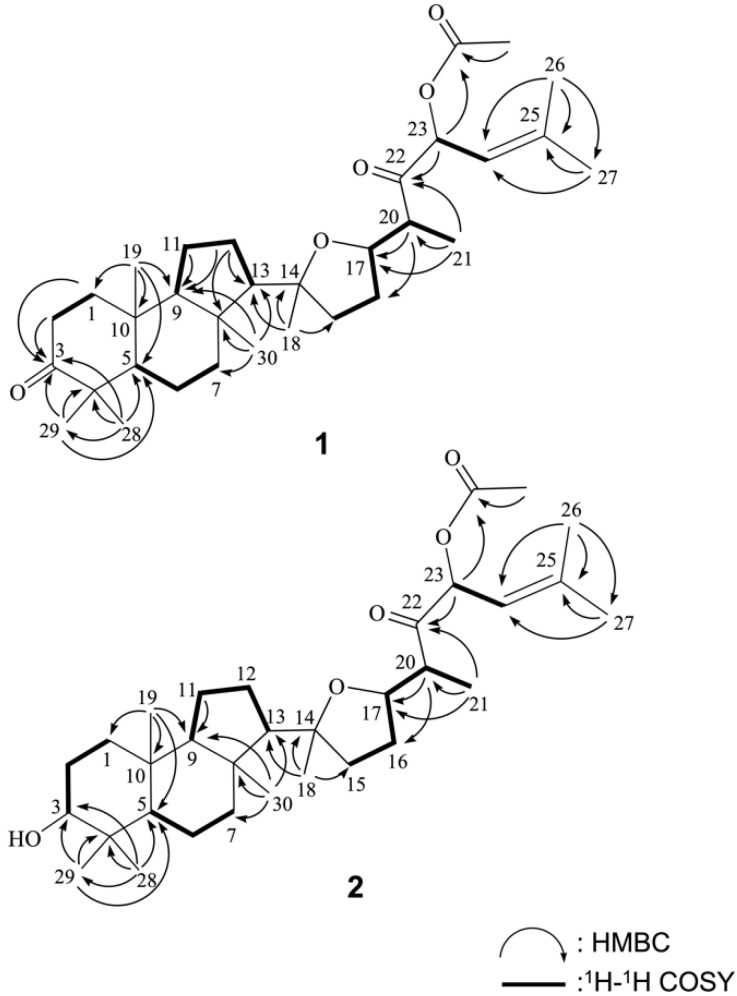
Selected ^1^H-^1^H COSY and HMBC correlations of **1** and **2**.

Based on the above results and with the assistance of ^1^H–^1^H COSY and HMBC experiments (Figure 2), the planar structure of **1** was determined. To establish the proton sequences in **1**, the ^1^H–^1^H COSY spectrum analysis established five proton sequences from H_2_-1 to H_2_-2, H-5 to H_2_-7, and H_2_-11 to H_2_-12, H_2_-15 to H-21, and H-23 to H-24. These data, together with the HMBC correlations (Figure 2) from H_2_-1 and H_2_-2 to C-3, H_2_-11 and H_2_-12 to C-9, H_2_-12 to C-8 and C-13, H_3_-19 to C-1, C-5, C-9 and C-10, H_3_-28 and H_3_-29 to C-3, C-4 and C-5 and H_3_-30 to C-7, C-8, C-9 and C-13 established the connectivity within the 6-membered (A), 6-membered (B), and 5-membered (C) rings.

Ring A of **1** was found to possess one ketone at C-3 and two methyl groups (C-28 and C-29), one methyl group (C-19), and one methyl groups (C-30) attached at C-4, C-8, and C-10, respectively. The key HMBC correlations suggested the connection of H_3_-18 to C-13, C-14, and C-15, H-20 to C-16 and C-17; H_3_-21 to C-17, C-20, and C-22; H-23 to C-22; H_3_-26 and H_3_-27 to C-24 and C-25. Thus, the side chain C-20 to C-27 was found to possess one double bond at C-24/C-25, one ketone group at C-22, two methyl groups at C-25, and one methyl group at C-20. One acetoxy group at C-23 was confirmed by the HMBC correlation between an oxymethine proton resonating at *δ*_H_ 6.09 (H-23) and the protons of an acetate methyl (*δ*_H_ 2.12) to the ester carbonyl carbon at *δ*_C_ 169.8. An ether linkage was proposed between C-14 and C-17 forming a tetrahydrofuran ring based on the degrees of unsaturation and molecular formula. Based on the above analysis, the gross structure of **1** was established unambiguously and named rhabdastin H following up the previous investigation of cytotoxic isomalabaricanes with oxygenated and olefinic side chains from the sponge *Rhabdastrella globostellata* [9].

**Figure 3:**
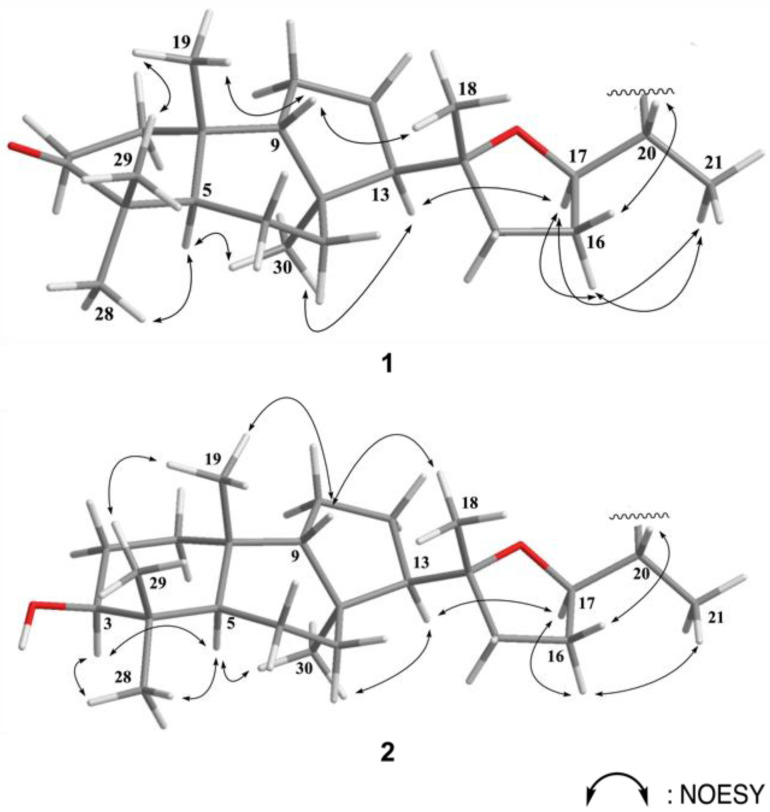
Selected NOESY correlations for **1** and **2**.

The relative configurations of the eight chiral centers at C-5, C-8, C-9, C-10, C-13, C-14, C-17, and C-20 in **1** were elucidated by the following NOE analysis (Figure 3). It was found that H_3_-19 (*δ*_H_ 0.79, s) showed NOE interaction with H-9 (*δ*_H_ 1.44, m) and H-9 with H_3_-18.

Since all naturally occurring isomalabaricanes displayed that H-5 is *trans* to Me-19, we assumed the *β*-orientation of H_3_-19. Thus, H_3_-18 and H-9 were suggested to be positioned on the *β*-face. One of the methyl groups (H_3_-29) at C-4, which showed a NOE correlation with H_3_-19, was *β*-oriented and the other one (H_3_-28) was *α*-oriented. The NOE correlations observed between H_3_-28/H-5, H-5/H_3_-30, H_3_-30/H-13, H-13/H-17, H-17/H_3_-21, and H-17/H-16 suggested *α*-orientation of H-5, H-13, H-17, H_3_-21, and H_3_-30. One of the methylene protons at C-16 (*δ*_H_ 1.97), which showed a NOE correlation with H-17 and H_3_-21, was assigned as H-16α and the other one (*δ*_H_ 1.58) as H-16*β*. Moreover, the detection of large proton coupling constants at H-17 (*J* = 13.5 Hz) and H-20 (*J* = 13.5 Hz) further supported their *trans* conformation in between. The observed NOE correlation between H-20 and H-16*β* suggested a *β*-orientation of H-20.

The HR-ESI-MS of rhabdastin I (**2**) showed a molecular ion peak at (*m*/*z* 539.3696 [M + Na]^+^) and the molecular formula C_32_H_52_O_5_ was suggested based on the HRESIMS and ^13^C NMR data. The IR spectrum of **2** showed the absorption of carbonyl groups (1745 and 1725 cm^−1^) and a hydroxy group (3436 cm^−1^). The ^1^H and ^13^C NMR spectroscopic data of **2** (Table 2) and **1** (Table 1) indicated similarity in structure except that the ketocarbonyl carbon (C-3) in **1** was replaced by a hydroxy group-bearing methine carbon in **2**. In the ^13^C NMR spectrum, the signal at *δ*_c_ 220.3 was replaced by a signal at *δ*_c_ 79.5, and in the ^1^H NMR spectrum, a signal at *δ*_H_ 3.24 (dd, *J* = 11.5, 6.5 Hz) was attributed to a hydroxy-bearing methine at C-3. H-3 showed an NOE correlation with H-5 (*δ*_H_ 1.54, m) suggesting a *β*-orientation of the hydroxy group at C-3. Therefore, **2** was identified as the 2*S*-hydroxy derivative of **1**.

**Figure 4:**
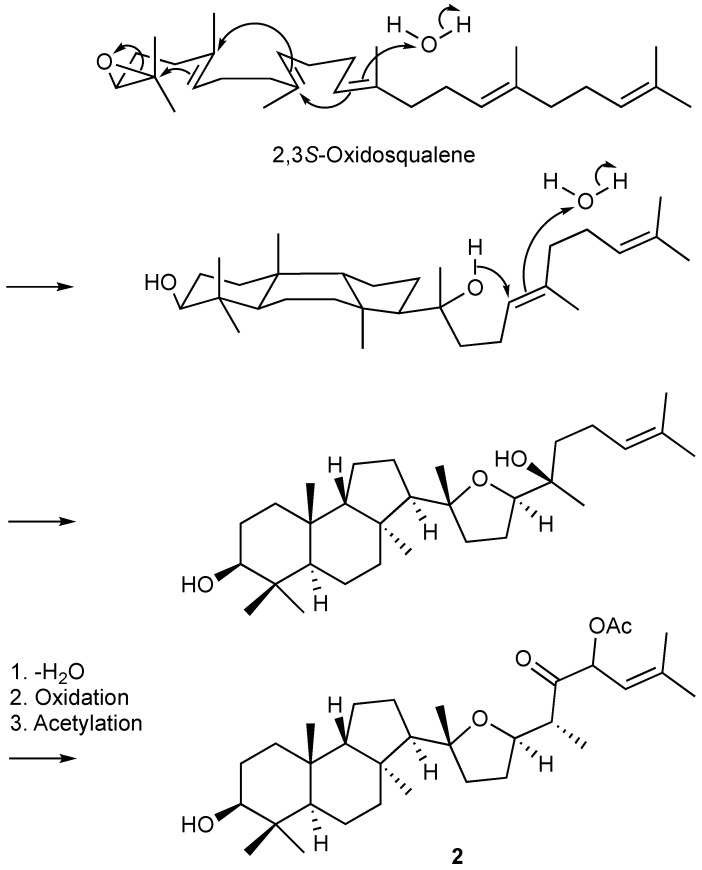
The plausible biosynthetic pathway of **2**.

The plausible biosynthesis route of the obtained isomalabaricanes presented in Figure 4. The main isomalabaricane 6/6/5 carbocyclic skeleton might be derived from 2,3*S*-oxidosqualene through hydroxylation and electrocyclizations. The attached tetrahydrofuran moiety and the subsequent side chain were suggested to go through a hydroxylation, an electrocyclization, dehydration, oxidation, and acetylation, forming the first identified isomalabaricane with tetrahydrofuran functionality.

To further clarify the anti-proliferative potential of the isolated compounds, four cancer cell lines (DLD-1: colorectal adenocarcinoma; T-47D: ductal carcinoma; Molt4: acute lymphoblastic leukemia; K562: chronic myelogenous leukemia) were used for MTT screening (Table 3). Compounds **1** and **2** were found to exhibit anti-proliferative activities against Molt4 and K562 leukemic cells with the IC_50_ value ranging from 9.81 to 16.54 μM.

## 3. Materials and Methods

### 3.1. General Experimental Procedures

Optical rotation spectra were recorded on a JASCO P-1010 polarimeter (JASCO, Tokyo, Japan). UV spectra were analyzed using JASCO UV-530 ultraviolet spectrophotometers. IR spectra were obtained on a Fourier-transform IR spectrophotometer Varian Digilab FTS 1000 (Varian Inc., Palo Alto, CA, USA). NMR spectra were obtained on a JEOL ECZ 400S or an ECZ 600R NMR (JEOL, Tokyo, Japan). HRESIMS data were collected on a Bruker APEX II instrument (Bruker Daltonik, Bremen, Germany). TLC was performed on Kieselgel 60 F_254_ (0.25 mm, Merck, Darmstadt, Germany) and/or RP-18 F_254_ (0.25 mm) coated plates and then visualized by spraying with 50% H_2_SO_4_ and heating on a hot plate. Silica gel 60 (Merck, 40−63 μm and 63−200 μm) were used for column chromatography. A Rheodyne 7725 injection port, a Hitachi L-2455 Photodiode Array Detector, and a Hitachi L-7100 pump (Hitachi, Tokyo, Japan), as well as a column Supelco Ascentis^®^ C-18 Cat #: 581343-U, were applied for HPLC chromatography. All methods were carried out following the relevant guidelines and regulations.

### 3.2. Animal Material

The specimen of the wild-type sponge *Rhabdastrella* sp. was collected by scuba diving at a depth about 3–5 m from Kenting, Pingtung, Taiwan in December 2017. The voucher specimen was deposited at −20 °C at the National Museum of Marine Biology and Aquarium, Taiwan (specimen No. 2017-1221-SP). Taxonomic identification was performed by Dr. Hsing-Hui Li using 18S DNA sequence and morphology determination.

### 3.3. Extraction and Isolation

*Rhabdastrella* sp. (500 g fresh weight) was collected and freeze-dried. The freeze-dried material (75 g, dry weight) was minced and extracted three times with a 1:1 mixture of methanol (MeOH) and dichloromethane (CH_2_Cl_2_). The crude extract was evaporated under reduced pressure to afford a residue (8 g), and the residue was subjected to a normal phase column chromatography on silica gel (70–230 mesh), using *n*-hexane, increasing polarity of *n*-hexane:EtOAc mixtures, and acetone to yield 13 fractions: L1 (eluted by *n*-hexane), L2 (eluted by *n*-hexane:EtOAc, 100:1), L3 (50:1), L4 (30:1), L5 (20:1), L6 (10:1), L7 (5:1), L8 (3:1), L9 (2:1), L10 (1:1), L11 (1:2), L12 (eluted by EtOAc) and L13 (eluted by acetone). L10 was further separated with silica gel (*n*-hexane:acetone 6:1) using normal phase HPLC to afford ten subfractions (L10-1 to L10-10). Subfraction L10-4 was then subjected to a reversed-phase HPLC (RP-HPLC) (MeOH:H_2_O, 85:15), yielding **1** (3.1 mg). Similarly, the subfraction L10-6 was purified by RP-HPLC (MeOH:H_2_O, 80:20) to provide **2** (8.5 mg).

Rhabdastin H (**1**): Colorless oil; ${\left[\alpha \right]}_{D}^{25}$ −47.1 (*c* 0.03, CHCl_3_); IR (ATR, CHCl_3_) *ν*_max_ 1742 and 1726 cm^–1^; ^1^H NMR data, see Table 1; HRESIMS *m*/*z* 537.3539 [M + Na]^+^ (calcd. 537.3550, see supplementary materials).

Rhabdastin I (**2**): Colorless oil; ${\left[\alpha \right]}_{D}^{25}$ −137.5 (*c* 0.02, CHCl_3_); IR (ATR, CHCl_3_) *ν*_max_ 3436, 1745, and 1725 cm^–1^; ^1^H NMR data, see Table 2; ESIMS *m*/*z* 539.3696 [M + Na]^+^ (calcd. 539.3707, see supplementary materials).

### 3.4. MTT Cell Proliferation Assay

MTT assay was used to examine the cellular proliferation of DLD-1 (colorectal adenocarcinoma), T-47D (ductal carcinoma), Molt4 (acute lymphoblastic leukemia), and K562 (chronic myelogenous leukemia) after 1 and 2 treatments. American Type Culture Collection (ATCC, Manassas, VA, USA) was the source for all cell lines. Briefly, cells at 1 × 10^5^ cells/mL were seeded at 96-well plates (150 μL/well) and incubated with several concentrations of compounds 1 and 2 for 24 h. After adding 50 μL MTT solution (1 mg/mL in PBS), the culture was incubated at 37 °C for 4 h, following which 200 μL DMSO was added to dissolve the formazan. The plate was then read on an ELISA microplate reader at 595 nm absorbance.

## 4. Conclusions

The current study highlighted the discovery of the first isomalabaricane derivatives with tetrahydrofuran moiety. Although the identified functionalities did not lead to a dramatic increase in the anti-proliferative properties, the chemical diversity of this class of triterpenes was enriched by these compounds with such unique structures.

## Figures and Tables

**Table 1:** ^1^H, ^13^C, ^1^H–^1^H COSY, and HMBC NMR data of **1**.

Position	*δ*_H_ (*J* in Hz) *^a^*	*δ*_C_ (Mult.) *^b^*	^1^H–^1^H COSY	HMBC
1	2.08 m; 1.46 m	31.9 (CH_2_) *^d^*	H-2	C-3
2	2.66 m; 2.30 m	33.7 (CH_2_)		C-3
3		220.3 (C)		
4		46.8 (C)		
5	2.23 m	45.7 (CH)	H-6	
6	1.45 m; 1.34 m	20.0 (CH_2_)	H-5, H-7	
7	2.15 m; 1.35 m	32.3 (CH_2_)		C-5, C-9
8		42.2 (C)		
9	1.44 m	52.9 (CH)	H-11	
10		35.0 (C)		
11	1.52 m; 1.30 m	21.4 (CH_2_)	H-9, H-12	C-9
12	1.85 m; 1.55 m	25.4 (CH_2_)	H-11, H-13	C-8, C-9, C-13
13	1.78 m	59.8 (CH)	H-12	
14		86.2 (C)		
15	1.80 m	38.6 (CH_2_)	H-16	
16	1.97 m; 1.58 m	31.0 (CH_2_)	H-17	
17	3.76 ddd (14.0, 8.5, 4.5) *^c^*	79.8 (CH)	H-20	
18	1.17 s	24.5 (CH_3_)		C-13, C-14, C-15
19	0.79 s	24.0 (CH_3_)		C-1, C-5, C-9, C-10
20	2.70 m	47.6 (CH)	H-17, H-21	C-16, C-17
21	0.95 d (7.0)	12.1 (CH_3_)	H-20	C-17, C-20, C-22
22		207.4 (C)		
23	6.09 d (10.0)	77.0 (CH)	H-24	OAc
24	5.11 d (10.0)	116.9 (CH)	H-23	
25		142.6 (C)		
26	1.81 s	26.0 (CH_3_)		C-24, C-25, C-27
27	1.89 s	19.0 (CH_3_)		C-24, C-25, C-26
28	1.02 s	19.3 (CH_3_)		C-3, C-4, C-5, C-29
29	1.06 s	29.3 (CH_3_)		C-3, C-4, C-5, C-28
30	1.09 s	31.8 (CH_3_)		C-7, C-8, C-9, C-13
OAc	2.12 s	20.9 (CH_3_)169.8 (C)		

*^a^* 500 MHz in CDCl_3_; *^b^* 125 MHz in CDCl_3_; *^c^ J* values (Hz) are given in parentheses; *^d^* Numbers of the attached protons were deduced by DEPT experiments.

**Table 2:** ^1^H, ^13^C, ^1^H–^1^H COSY, and HMBC NMR data of **2**.

Position	*δ*_H_ (*J* in Hz) *^a^*	*δ*_C_ (Mult.) *^b^*	^1^H–^1^H COSY	HMBC
1	1.48 m; 1.41 m	33.9 (CH_2_) *^d^*	H-2	
2	1.73 m; 1.61 m	29.2 (CH_2_)	H-3	
3	3.24 dd (11.5,6.5)	79.5 (CH)		
4		39.0 (C)		
5	1.54 m	46.6 (CH)	H-6	
6	1.64 m; 1.38 m	18.5 (CH_2_)	H-5, H-7	
7	2.04 m; 1.35 m	32.9 (CH_2_)		
8		42.0 (C)		
9	1.42 m	55.0 (CH)	H-11	
10		35.6 (C)		
11	1.49 m; 1.30 m	21.2 (CH_2_)	H-9. H-12	C-9
12	1.82 m; 1.54 m	25.0 (CH_2_)	H-11, H-13	
13	1.78 m	59.9 (CH)	H-12	
14		86.2 (C)		
15	1.80 m; 1.75 m	38.8 (CH_2_)	H-16	
16	1.98 m; 1.60 m	31.1 (CH_2_)	H-17	
17	3.75 ddd (13.5, 8.5, 4.5) *^c^*	79.5 (CH)	H-20	
18	1.16 s	24.5 (CH_3_)		C-13, C-14, C-15
19	0.95 s	23.1 (CH_3_)		C-1, C-5, C-9, C-10
20	2.69 ddd (13.5, 8.5, 1.5)	47.5 (CH)	H-17, H-21	C-16, C-17
21	0.94 d (6.0)	12.1 (CH_3_)	H-20	C-17, C-20, C-22
22		207.4 (C)		
23	6.10 d (10.0)	78.7 (CH)	H-24	C-22, C-24, OAc
24	5.11 d (10.0)	116.9 (CH)	H-23	
25		142.6 (C)		
26	1.81 s	26.0 (CH_3_)		C-24, C-25, C-27
27	1.89 s	19.0 (CH_3_)		C-24, C-25, C-26
28	0.98 s	15.9 (CH_3_)		C-3, C-4, C-5, C-29
29	0.78 s	29.1 (CH_3_)		C-3, C-4, C-5, C-28
30	1.04 s	31.6 (CH_3_)		C-7, C-8, C-9, C-14
OAc	2.10 s	20.9 (CH_3_)169.8 (C)		

*^a^* 500 MHz in CDCl_3_; *^b^* 125 MHz in CDCl_3_; *^c^ J* values (Hz) are given in parentheses; *^d^* Numbers of the attached protons were deduced by DEPT experiments.

**Table 3:** Anti-proliferative activities of compounds **1** and **2**.

Compounds	Cell Lines (IC_50_ μM)			
	DLD-1	T-47D	Molt4	K562
**1**	– *^a^*	– *^a^*	16.54	11.71
**2**	– *^a^*	17.48	11.03	9.81
Doxorubicin *^b^*	0.42	0.18	0.02	0.28

*^a^* IC_50_ > 20 μM; *^b^* positive control.

## Data Availability

The data presented in this study are available on request from the corresponding author.

## Supplementary Materials

The following are available online at https://www.mdpi.com/article/10.3390/md19040206/s1, ESIMS, HRESIMS, IR, 1D, 2D, and DEPT NMR spectra of **1**–**2**.
