# Supplementary material for: Isomalabaricane Triterpenes from the Marine Sponge Rhabdastrella sp
Source: Mar Drugs. 2021 Apr 6;19(4):206. doi: 10.3390/md19040206 (PMC8067365; doi:10.3390/md19040206)
Supplement: Supplementary file 1 [file marinedrugs-19-00206-s001.pdf]

SUPPORTING INFORMATION

Article

Isomalabaricane Triterpenes from the Marine Sponge *Rhabdastrella* sp.

Kuei-Hung Lai [1,2,3,*], Zheng-Hao Huang [4,5], Mohamed El-Shazly [6,7], Bo-Rong Peng [4,5], Wen-Chi Wei [8,*] and Jui-Hsin Su [4,5,*]

Table of Contents

Figure S1. IR spectrum of **1** --- 2
Figure S2. ESIMS spectrum of **1** -- 2
Figure S3. HRESIMS spectrum of **1** -- 3
Figure S4. ^{1}H NMR (500 MHz, $CDCl_3$) spectrum of **1** ----------------------------------- 3
Figure S5. ^{1}H NMR (500 MHz, $CDCl_3$) spectrum of **1** (Partial enlarged view) ---------4
Figure S6. ^{13}C NMR (125 MHz, $CDCl_3$) spectrum of **1** ---------------------------------- 4
Figure S7. ^{13}C NMR (125 MHz, $CDCl_3$) spectrum of **1** (Partial enlarged view) -------- 5
Figure S8. DEPT spectrum of **1** --- 5
Figure S9. DEPT spectrum of **1** (Partial enlarged view) ----------------------------------- 6
Figure S10. HSQC spectrum of **1** --- 6
Figure S11. HSQC spectrum of **1** (Partial enlarged view) -------------------------------- 7
Figure S12. COSY spectrum of **1** --- 7
Figure S13. COSY spectrum of **1** (Partial enlarged view) --------------------------------- 8
Figure S14. HMBC spectrum of **1** -- 8
Figure S15. HMBC spectrum of **1** (Partial enlarged view) -------------------------------- 9
Figure S16. NOESY spectrum of **1** --- 9
Figure S17. IR spectrum of **2** --- 10
Figure S18. ESIMS spectrum of **2** -- 10
Figure S19. HRESIMS spectrum of **2** -- 11
Figure S20. ^{1}H NMR (500 MHz, $CDCl_3$) spectrum of **2** -------------------------------- 11
Figure S21. ^{1}H NMR (500 MHz, $CDCl_3$) spectrum of **2** (Partial enlarged view) ------ 12
Figure S22. ^{13}C NMR (125 MHz, $CDCl_3$) spectrum of **2** ------------------------------- 12
Figure S23. ^{13}C NMR (125 MHz, $CDCl_3$) spectrum of **2** (Partial enlarged view) ----- 13
Figure S24. DEPT spectrum of **2** -- 13
Figure S25. DEPT spectrum of **2** (Partial enlarged view) -------------------------------- 14
Figure S26. HSQC spectrum of **2** --- 14
Figure S27. HSQC spectrum of **2** (Partial enlarged view) -------------------------------- 15
Figure S28. COSY spectrum of **2** --- 15
Figure S29. COSY spectrum of **2** (Partial enlarged view) -------------------------------- 16
Figure S30. HMBC spectrum of **2** -- 16
Figure S31. HMBC spectrum of **2** (Partial enlarged view) ------------------------------- 17
Figure S32. NOESY spectrum of **2** --- 17

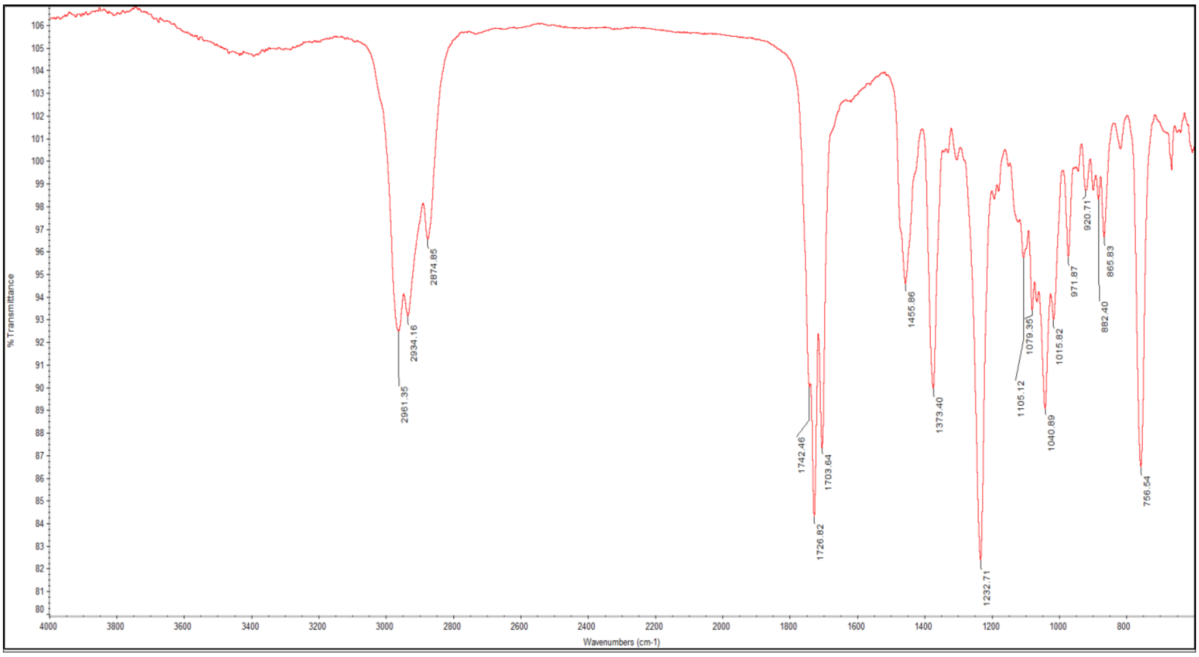

Figure S1. IR spectrum of **1**

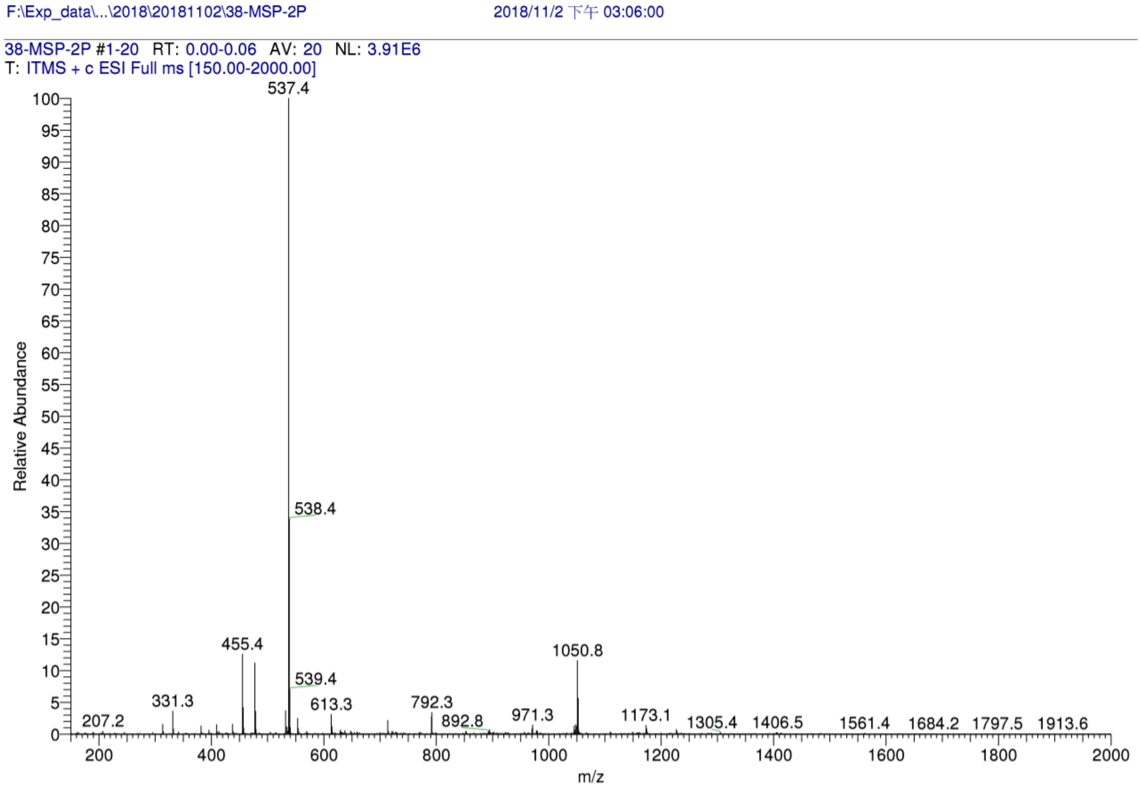

Figure S2. ESIMS spectrum of **1**

F:\Exp_data\...\20181102\35-MSP-2P-H 2018/11/2 下午 03:03:26

35-MSP-2P-H#1-20 RT: 0.00-0.27 AV: 20
T: FTMS + p ESI Full ms [150.00-2000.00]
m/z= 537.2468-537.4527

Isotope	Min	Max
C-12	0	32
H-1	0	51
O-16	0	5
Na-23	0	1

Charge 1
Mass tolerance 1000.00 ppm
Nitrogen rule not used
RDB equiv -1.00-100.00
max results 1

m/z	Intensity	Relative	Theo. Mass	Delta (ppm)	Composition
537.3539	48065596.0	100.00	537.3550	-2.09	$C_{32}H_{50}O_5Na$

Figure S3. HRESIMS spectrum of **1**

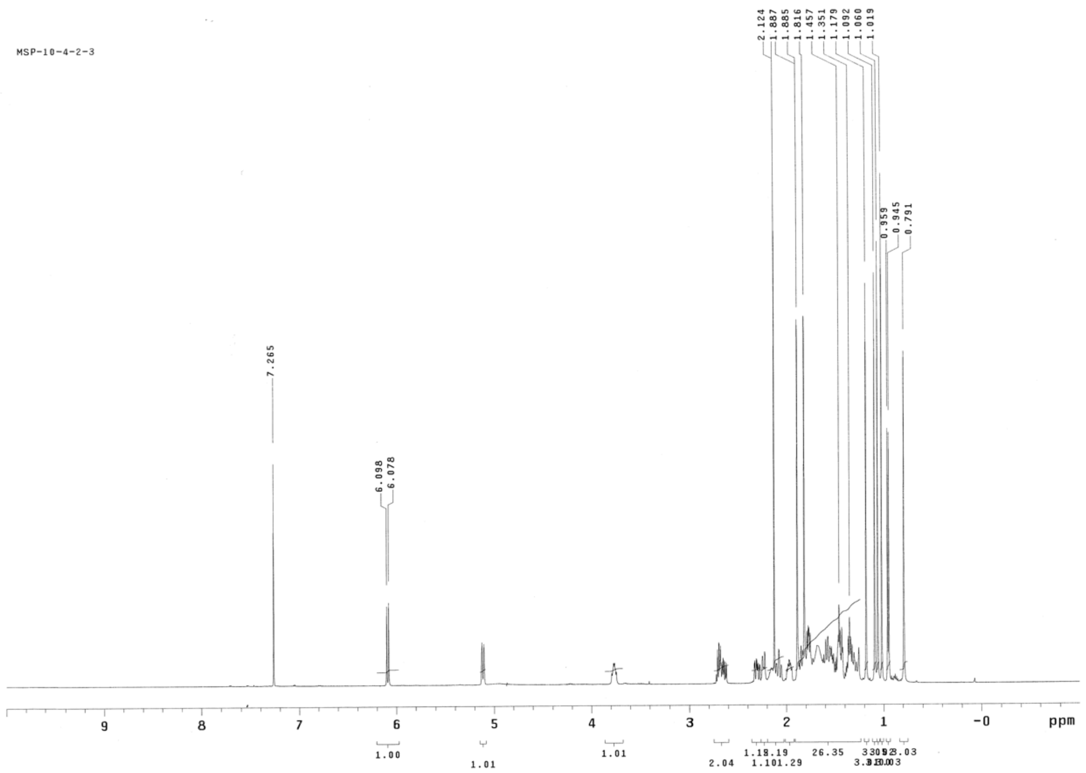

Figure S4. ^{1}H NMR (500 MHz, $CDCl_3$) spectrum of **1**

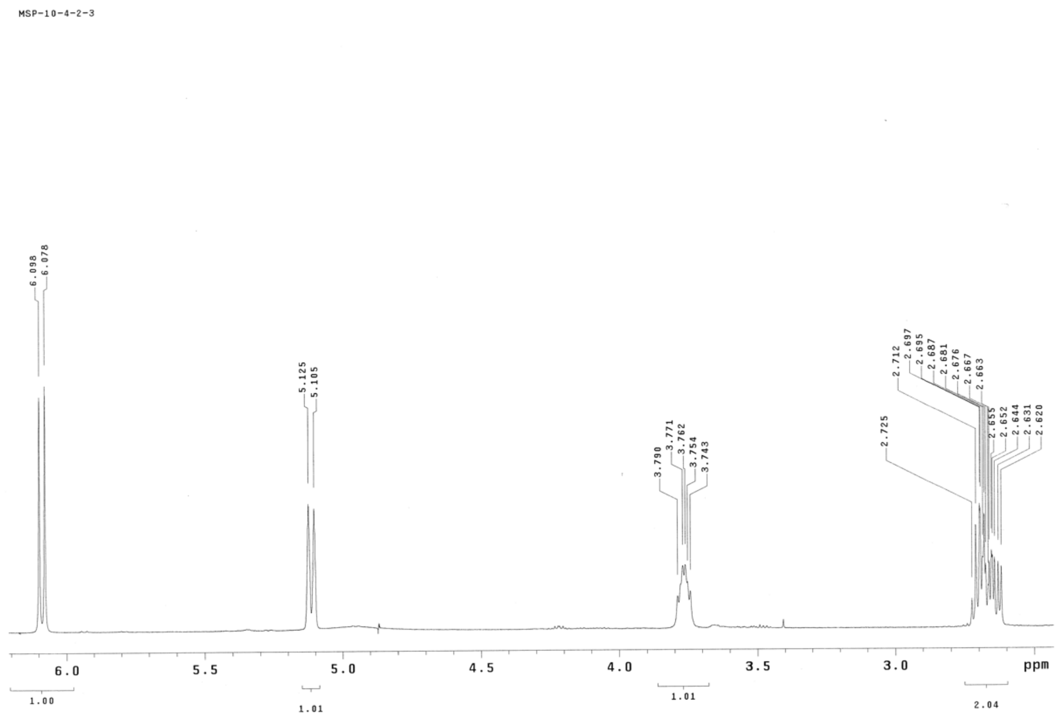

Figure S5. ^{1}H NMR (500 MHz, $CDCl_3$) spectrum of **1** (Partial enlarged view)

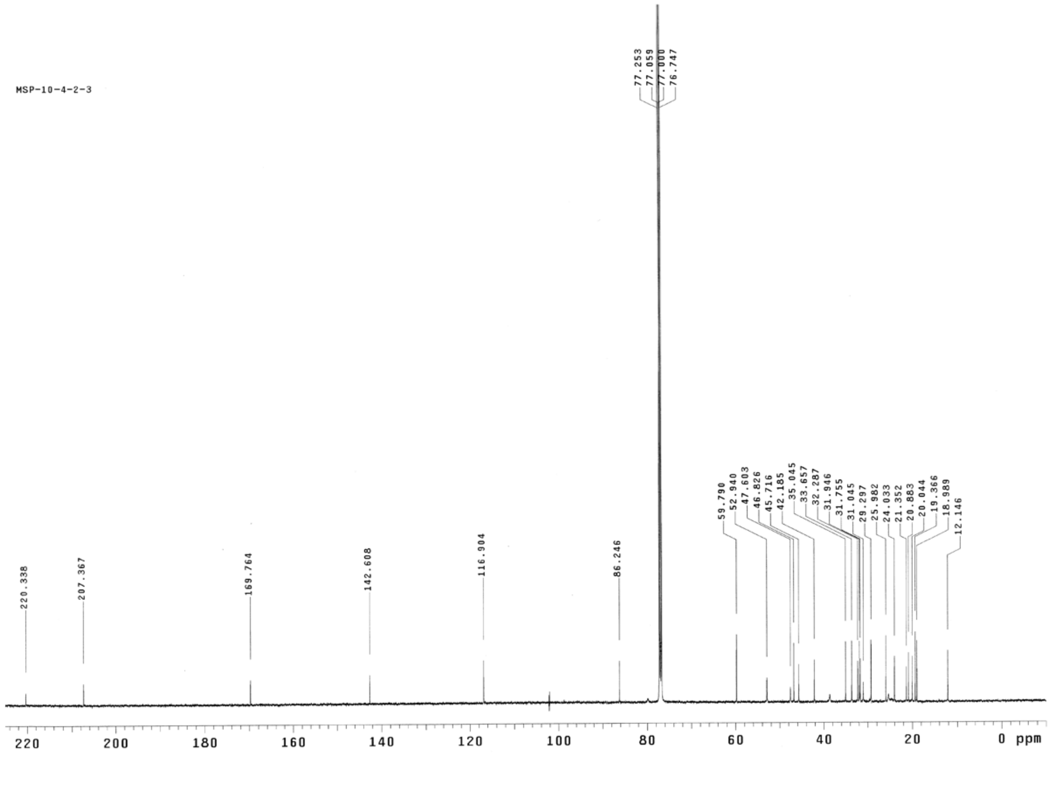

Figure S6. ^{13}C NMR (125 MHz, $CDCl_3$) spectrum of **1**

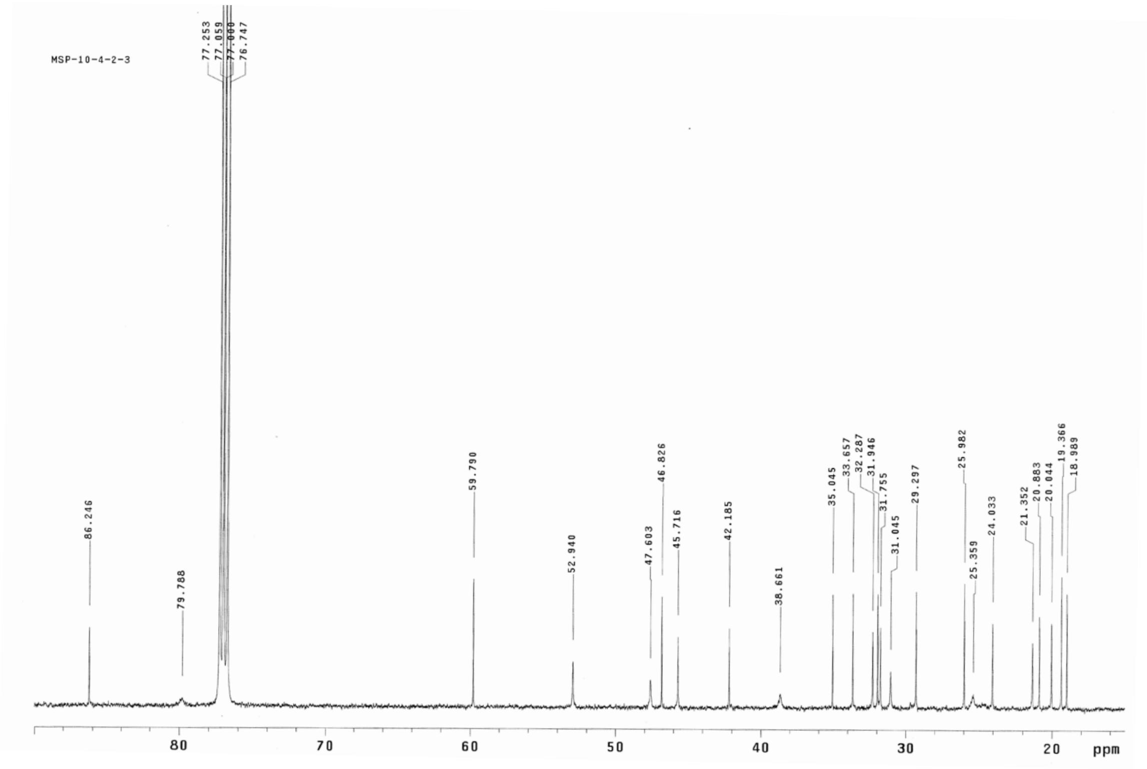

Figure S7. ^{13}C NMR (125 MHz, $CDCl_3$) spectrum of **1** (Partial enlarged view)

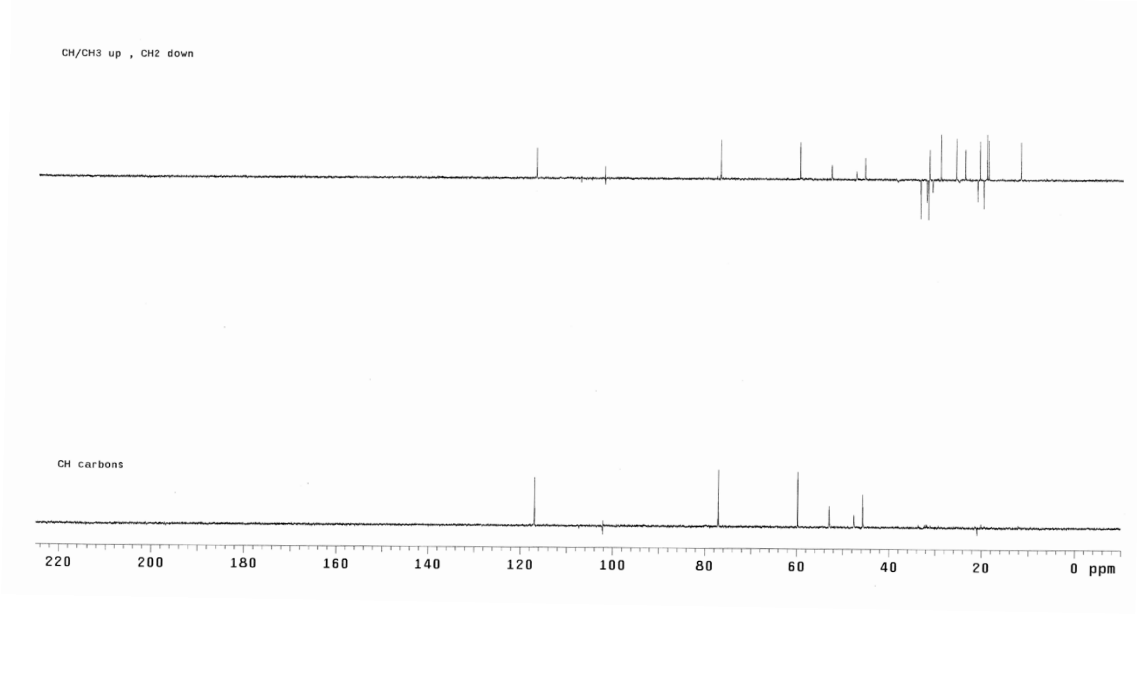

Figure S8. DEPT spectrum of **1**

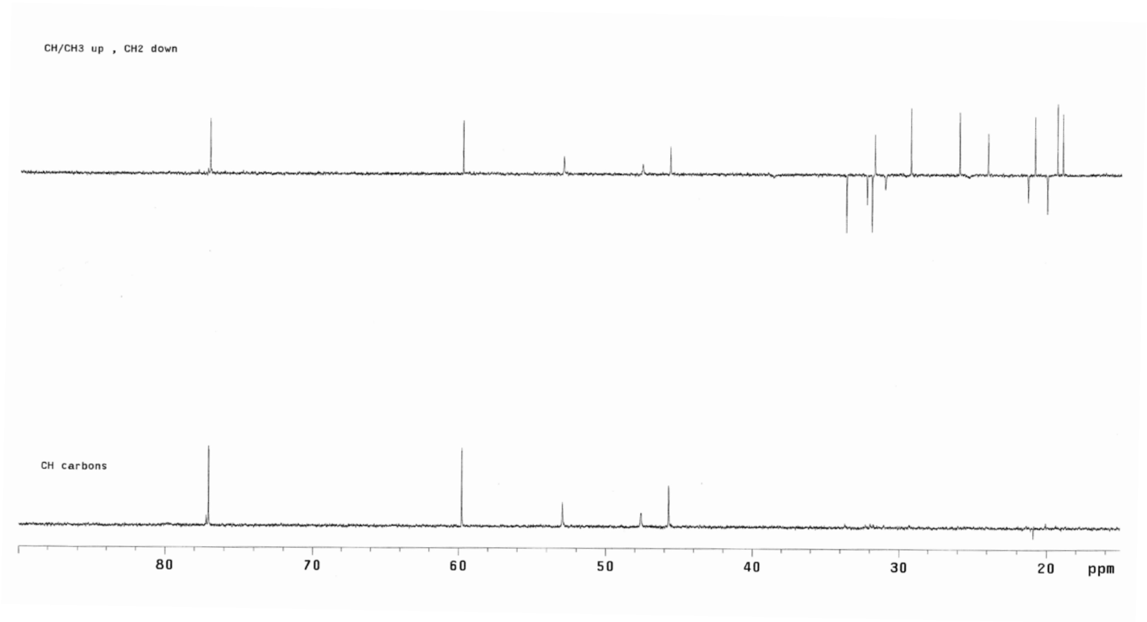

Figure S9. DEPT spectrum of **1** (Partial enlarged view)

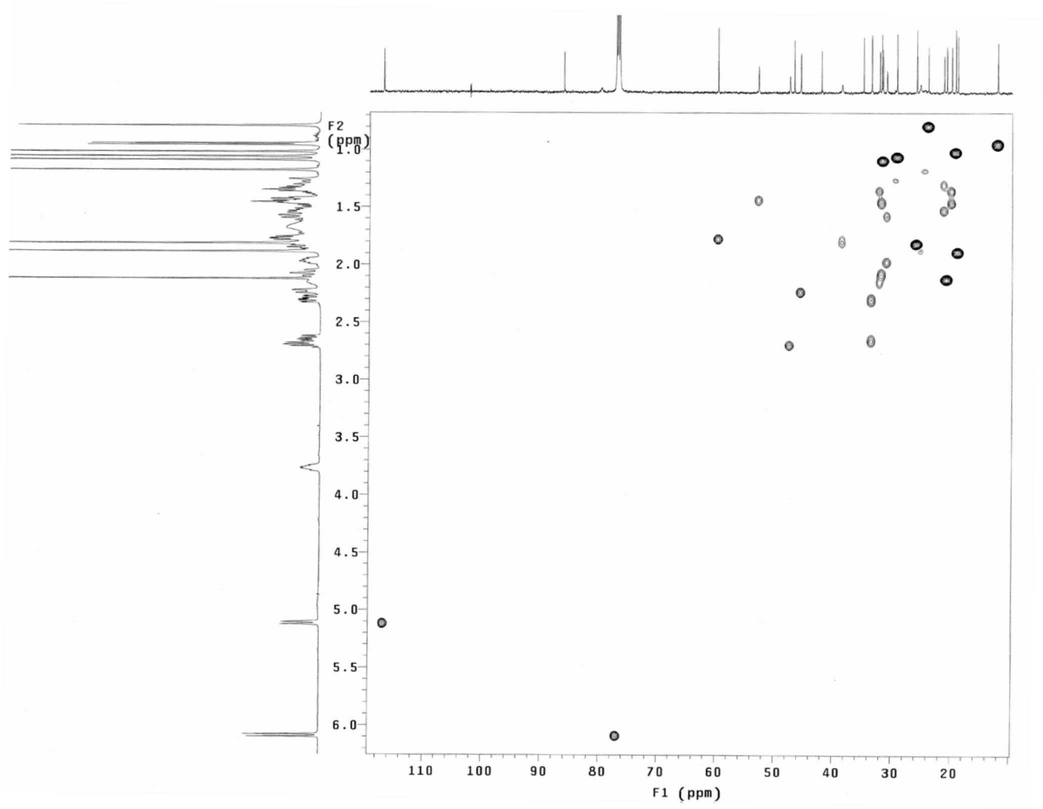

Figure S10. HSQC spectrum of **1**

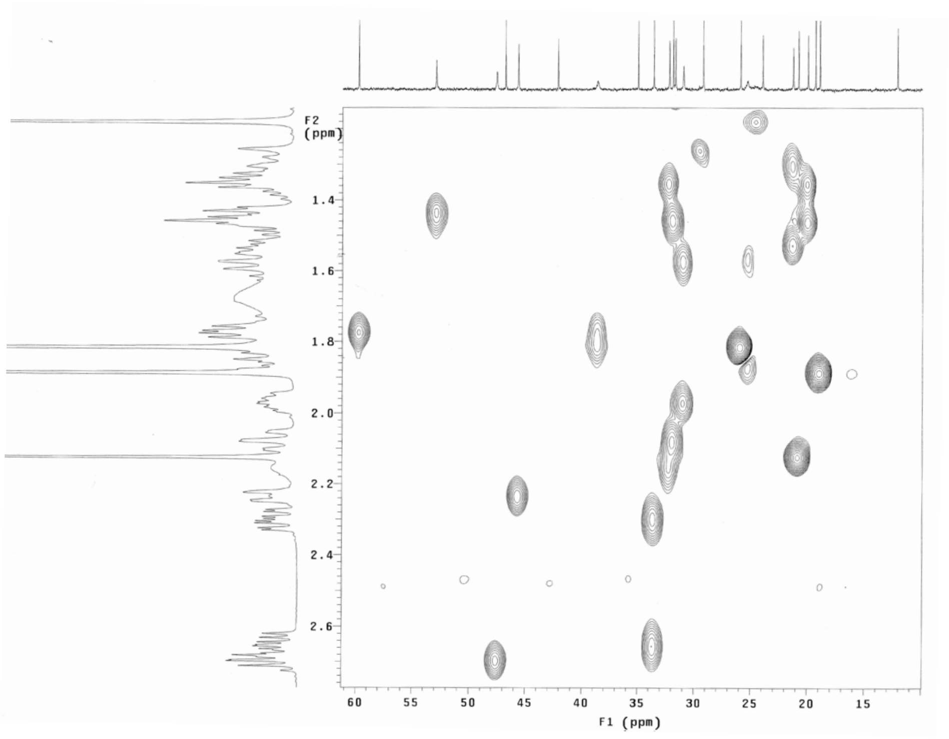

Figure S11. HSQC spectrum of **1** (Partial enlarged view)

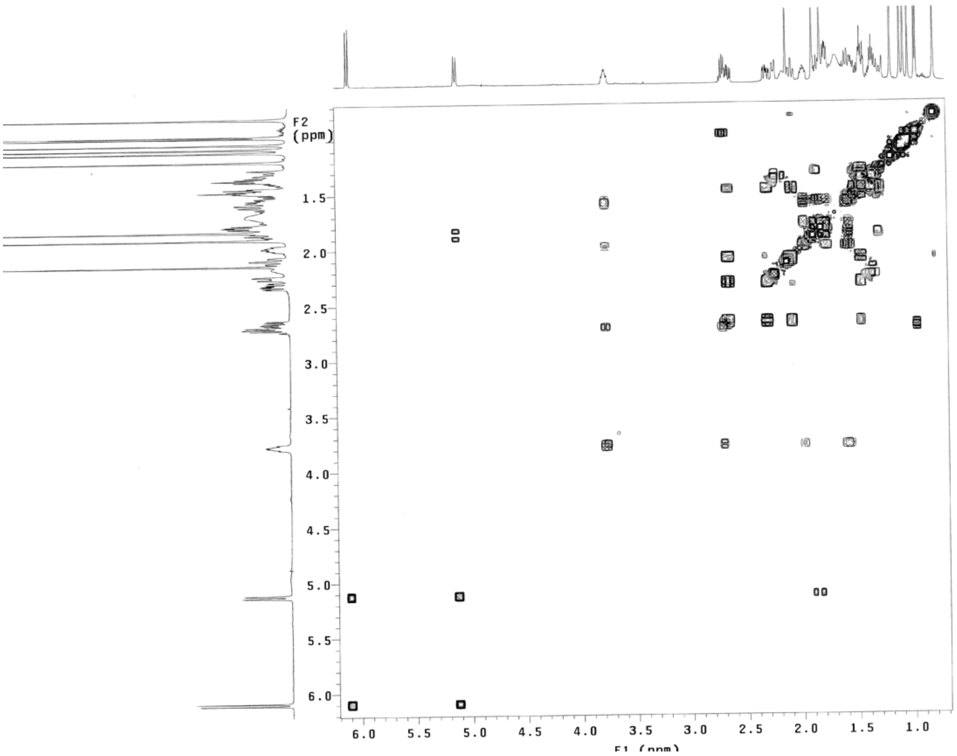

Figure S12. COSY spectrum of **1**

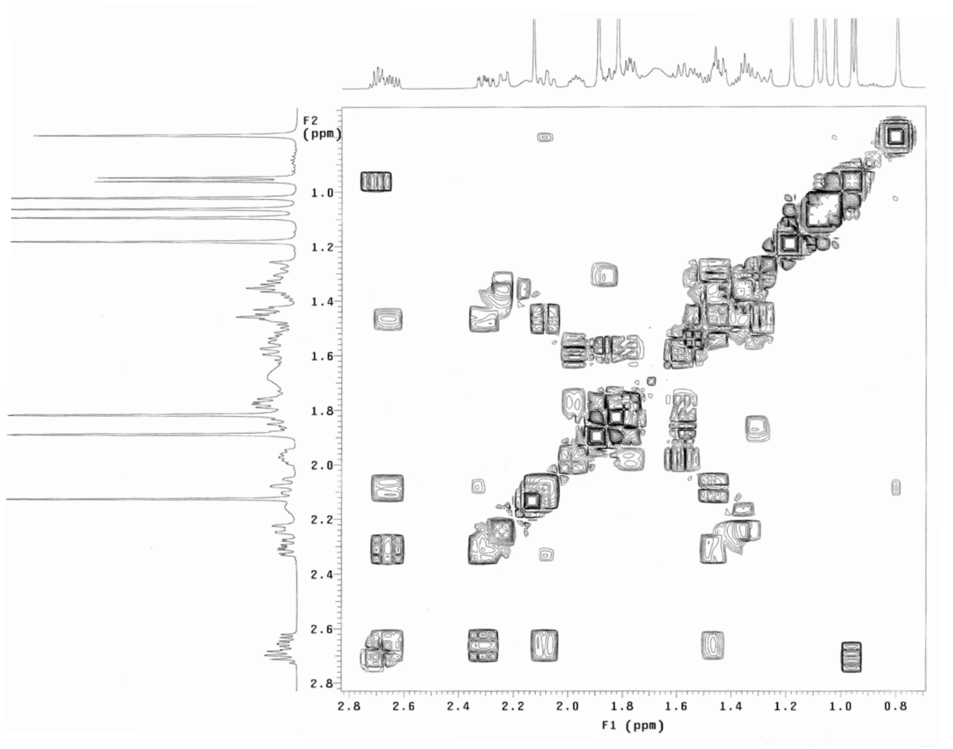

Figure S13. COSY spectrum of **1** (Partial enlarged view)

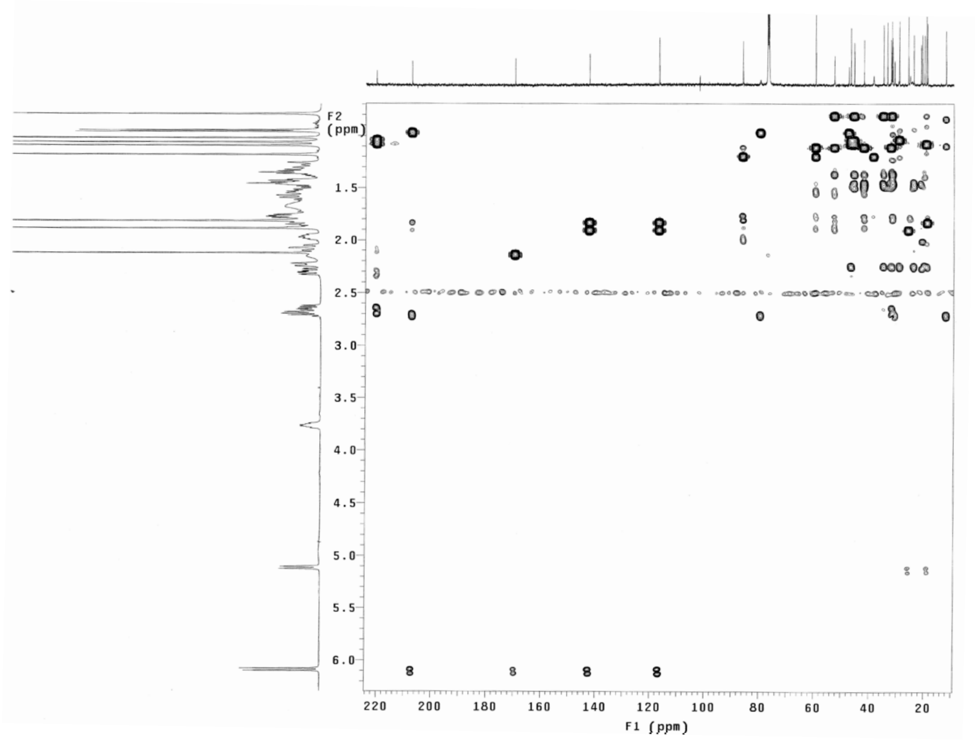

Figure S14. HMBC spectrum of **1**

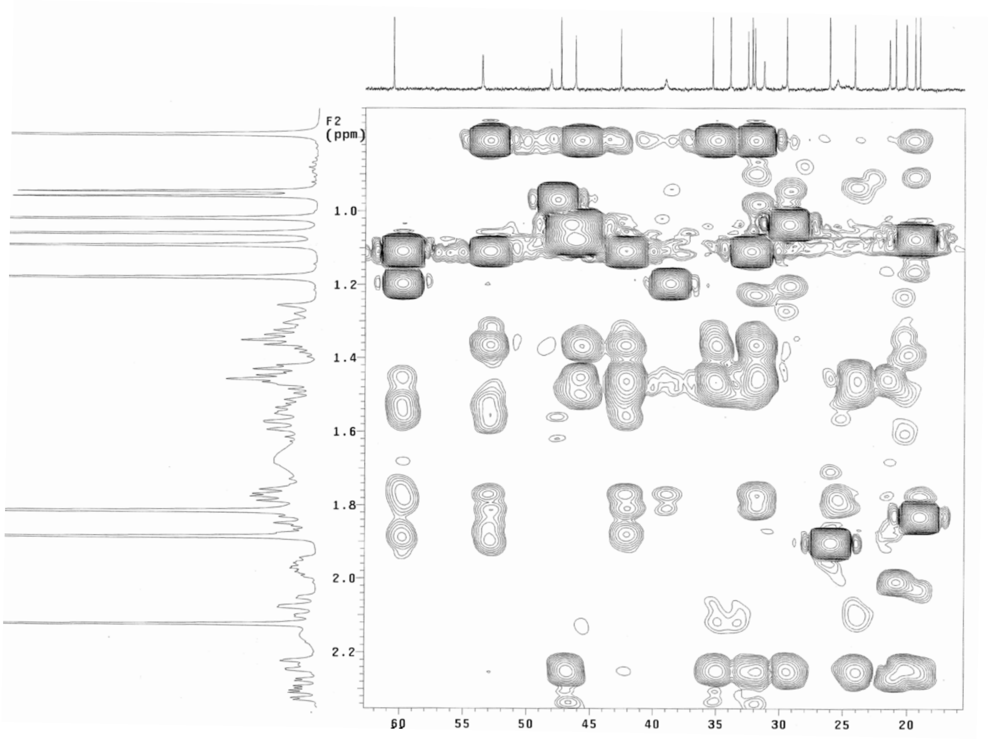

Figure S15. HMBC spectrum of **1** (Partial enlarged view)

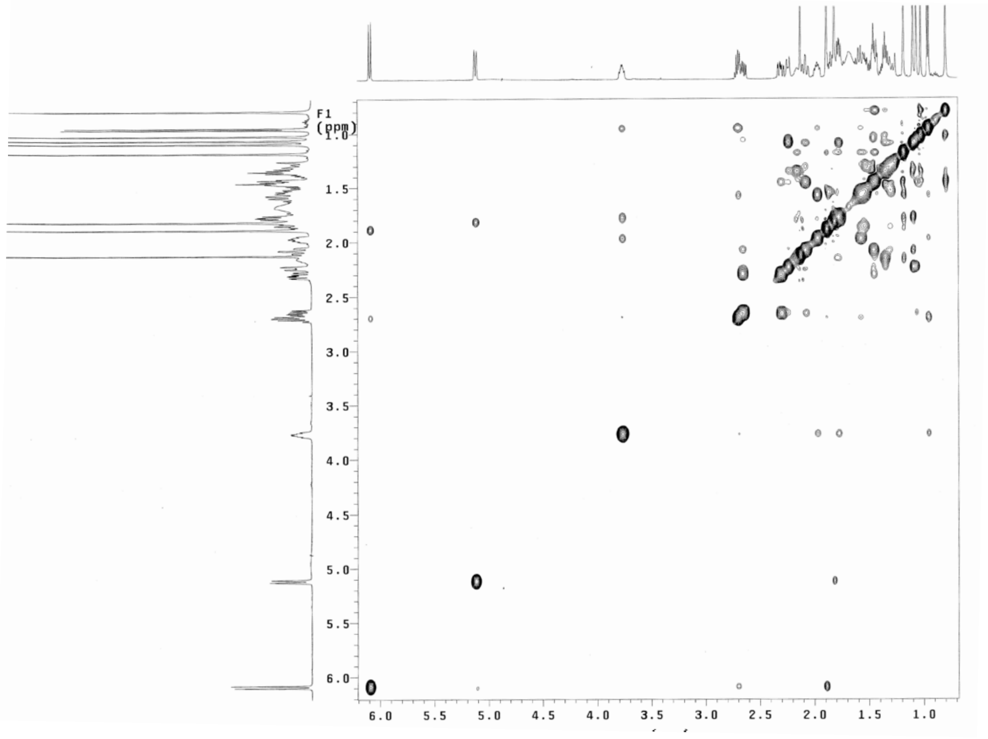

Figure S16. NOESY spectrum of **1**

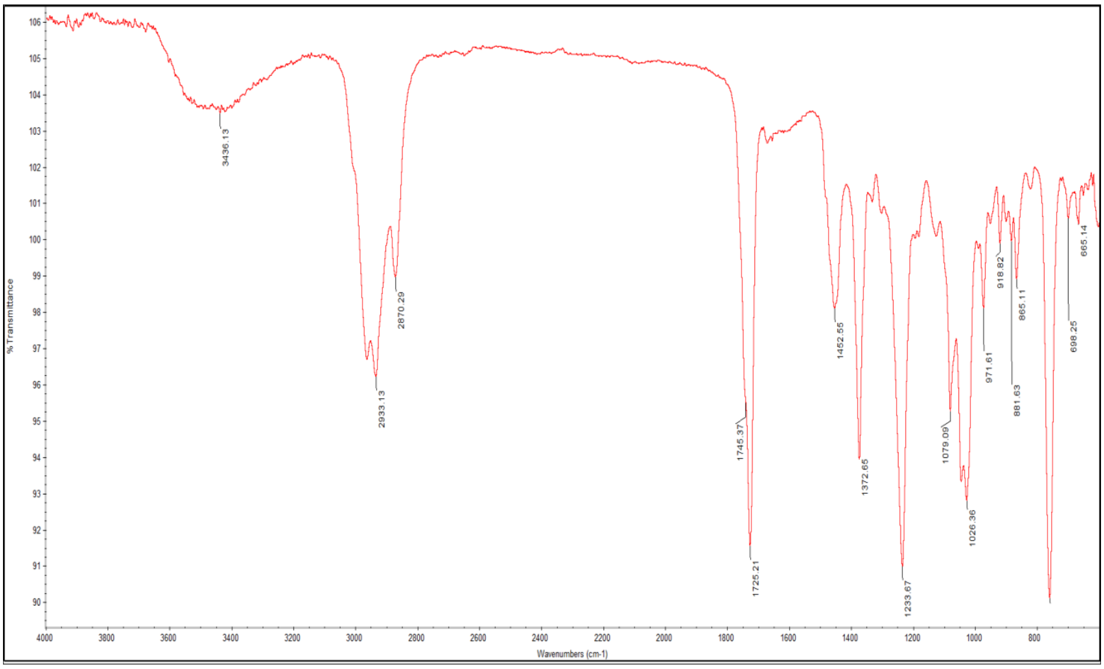

Figure S17. IR spectrum of **2**

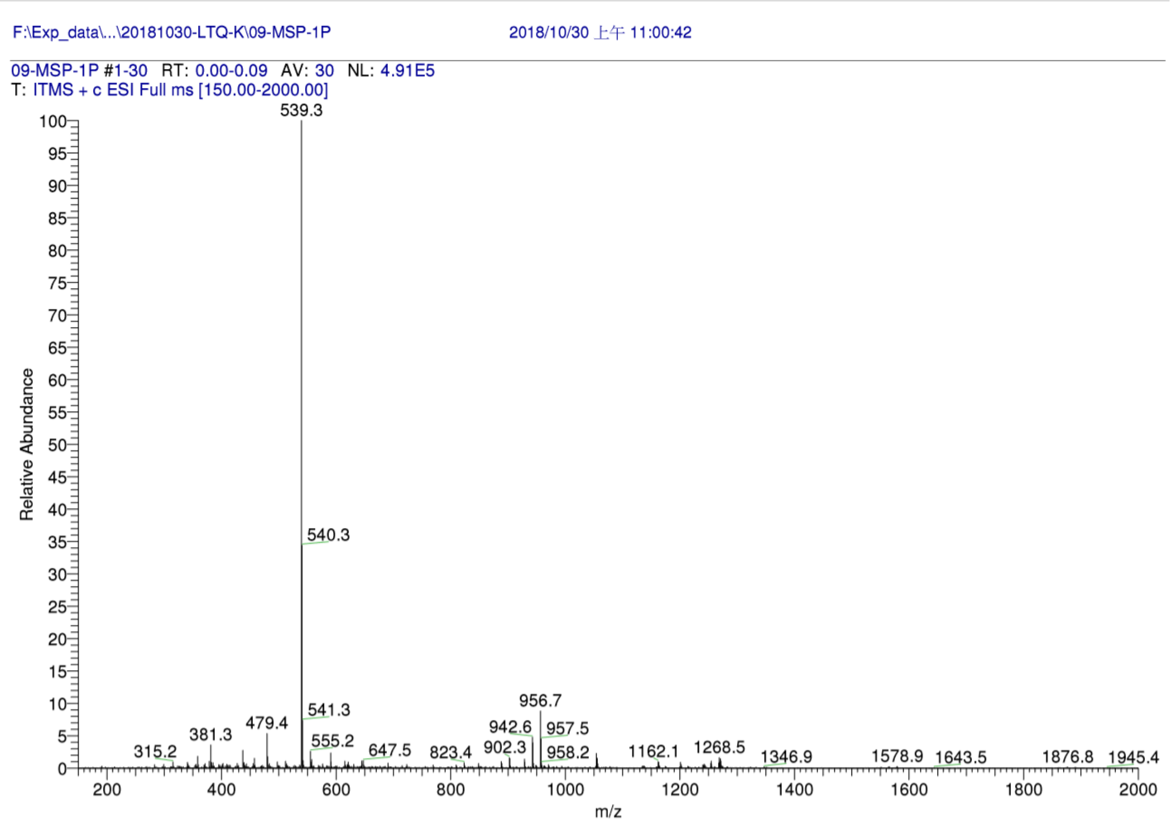

Figure S18. ESIMS spectrum of **2**

F:\Exp_data\...\20181102\45-MSP-1P-H 2018/11/2 下午 03:20:57

45-MSP-1P-H#1-20 RT: 0.01-0.28 AV: 20
T: FTMS + p ESI Full ms [150.00-2000.00]
m/z= 539.0505-539.6632

Isotope	Min	Max
C-12	0	32
H-1	0	53
O-16	0	5
Na-23	0	1

Charge 1
Mass tolerance 1000.00 ppm
Nitrogen rule not used
RDB equiv -1.00-100.00
max results 1

m/z	Intensity	Relative	Theo. Mass	Delta (ppm)	Composition
539.3696	55447880.0	100.00	539.3707	-2.04	$C_{32}H_{52}O_5Na$

Figure S19. HRESIMS spectrum of **2**

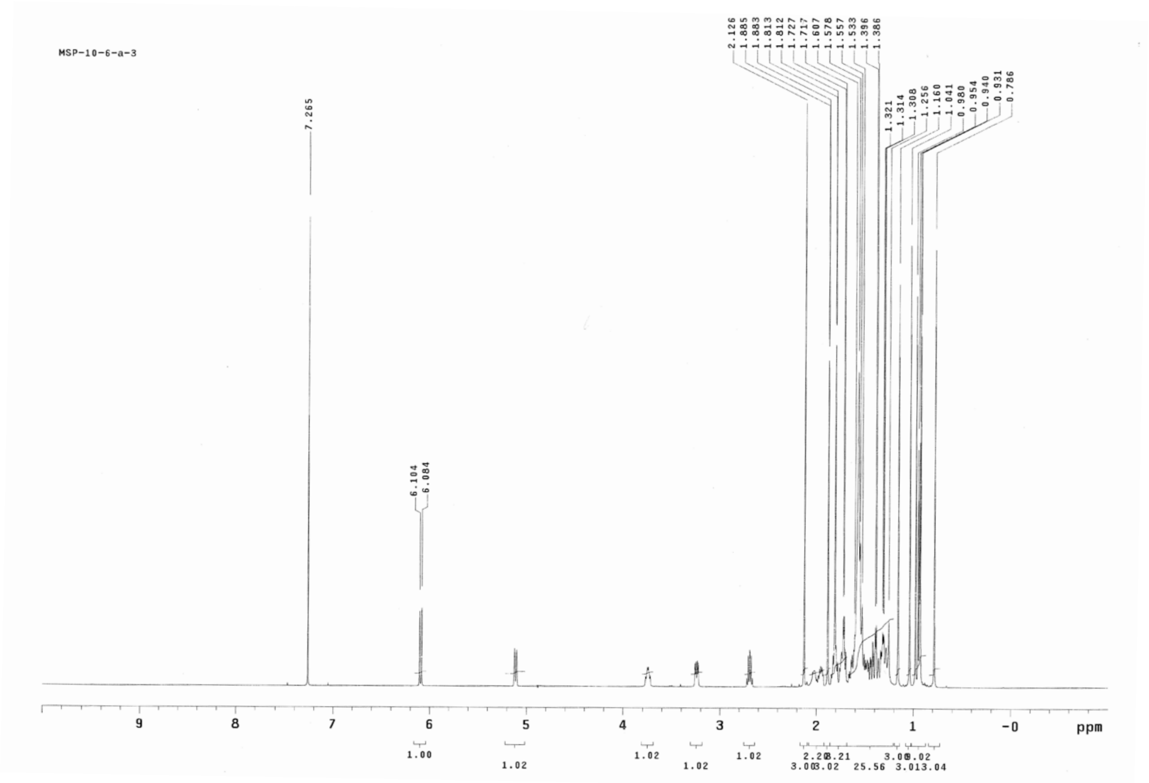

Figure S20. ^{1}H NMR (500 MHz, $CDCl_3$) spectrum of **2**

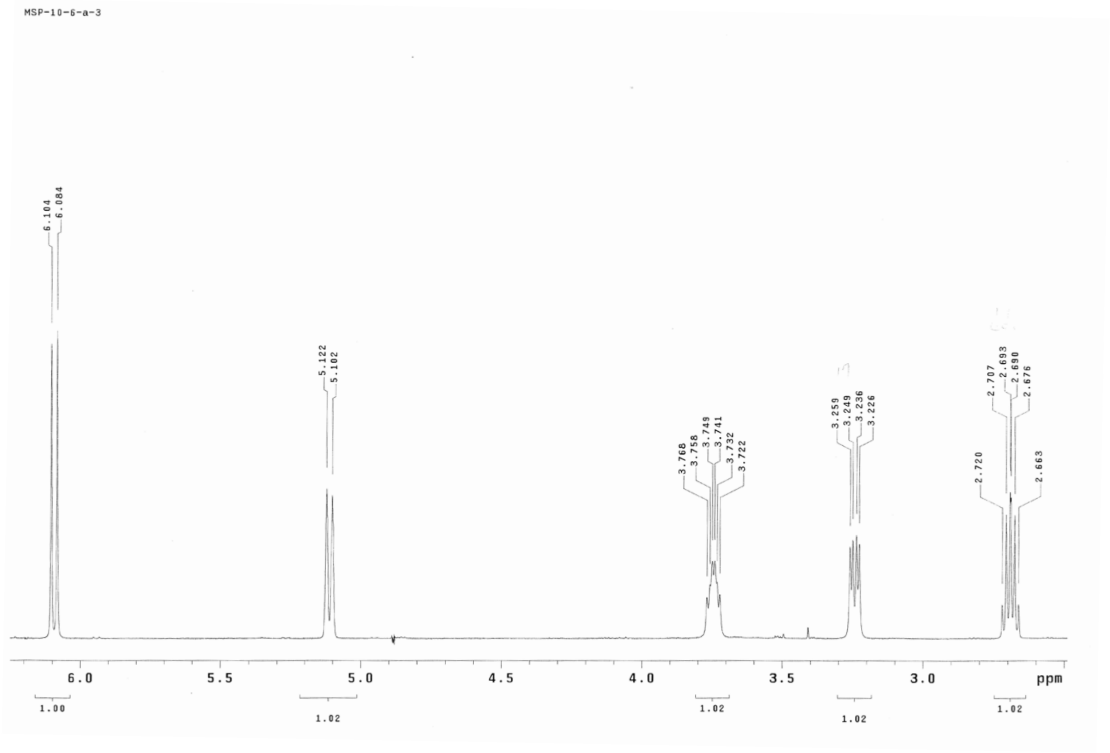

Figure S21. ^{1}H NMR (500 MHz, $CDCl_3$) spectrum of **2** (Partial enlarged view)

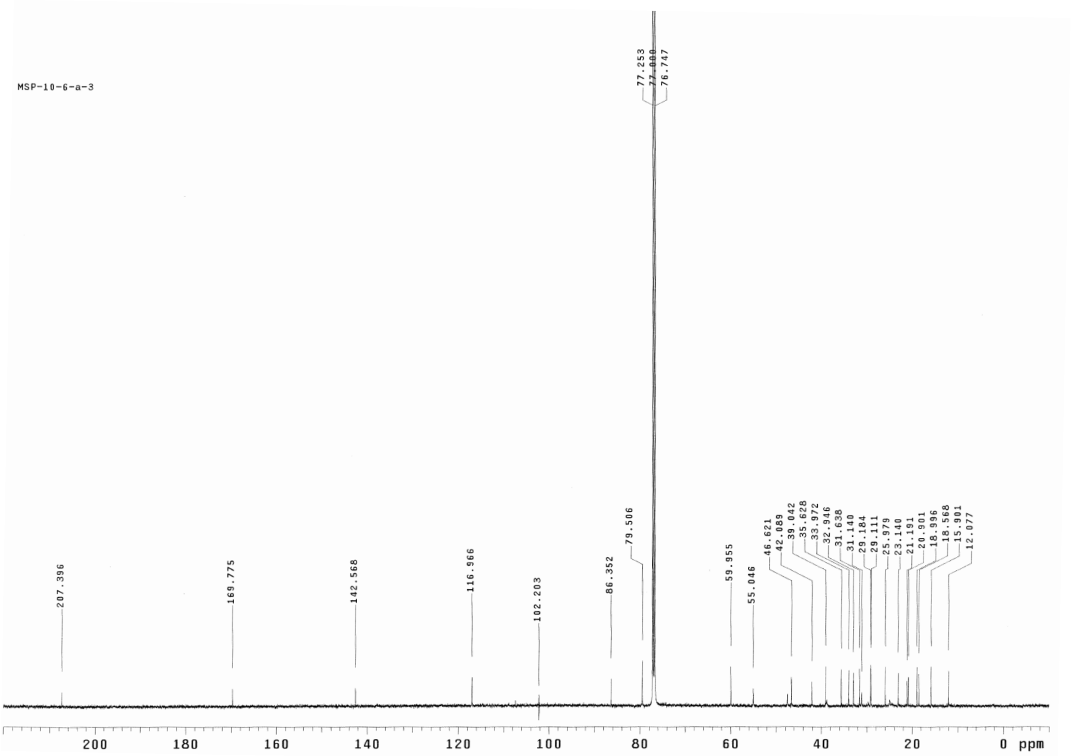

Figure S22. ^{13}C NMR (125 MHz, $CDCl_3$) spectrum of **2**

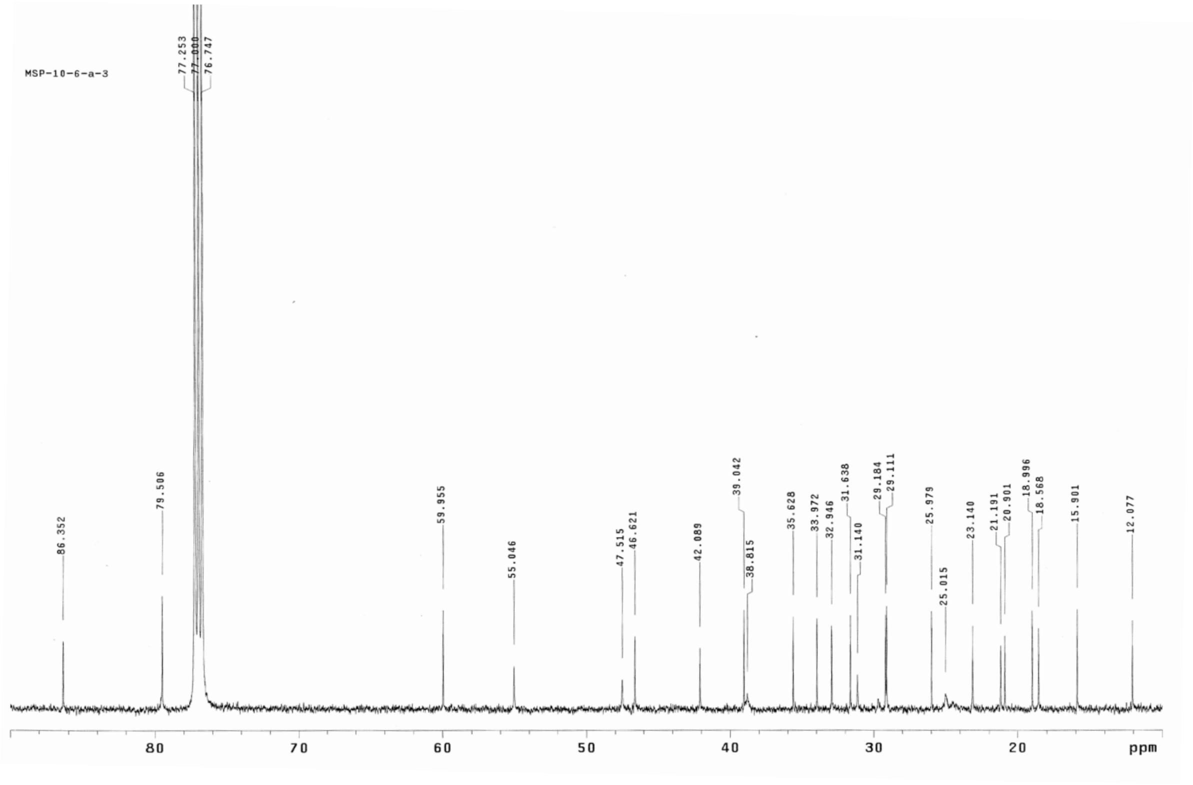

Figure S23. ^{13}C NMR (125 MHz, $CDCl_3$) spectrum of **2** (Partial enlarged view)

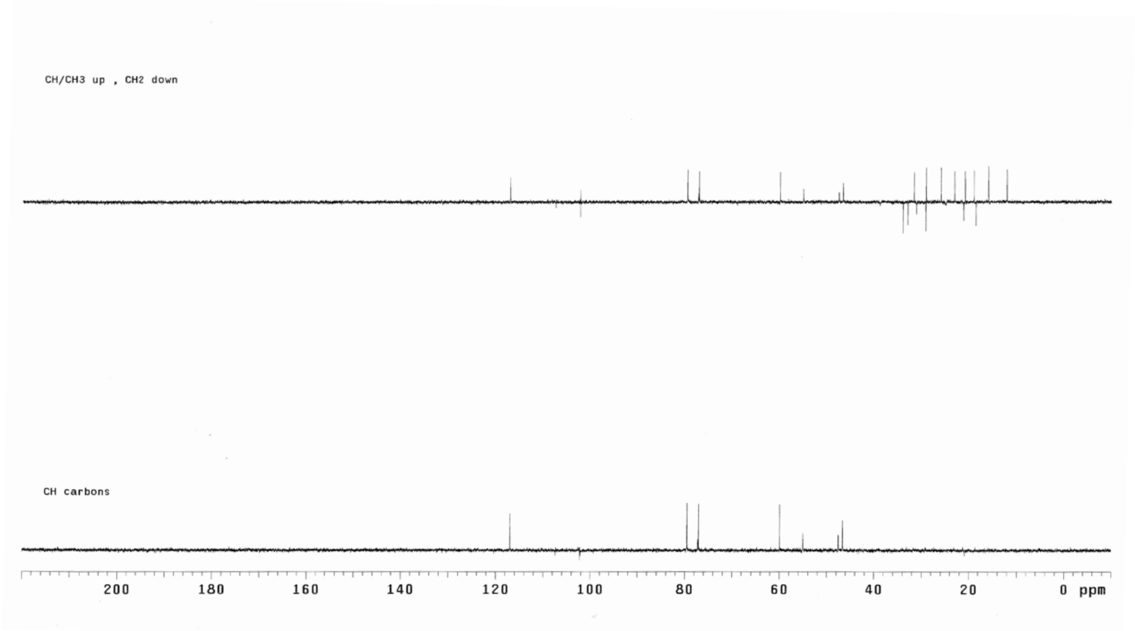

Figure S24. DEPT spectrum of **2**

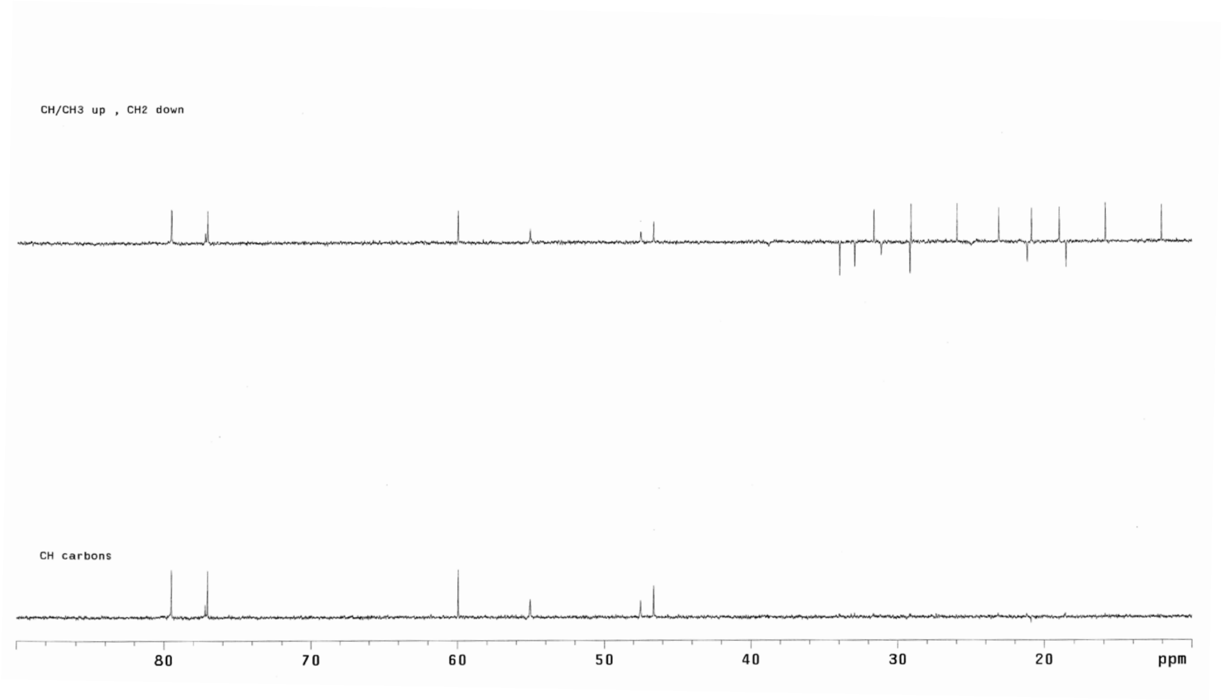

Figure S25. DEPT spectrum of **2** (Partial enlarged view)

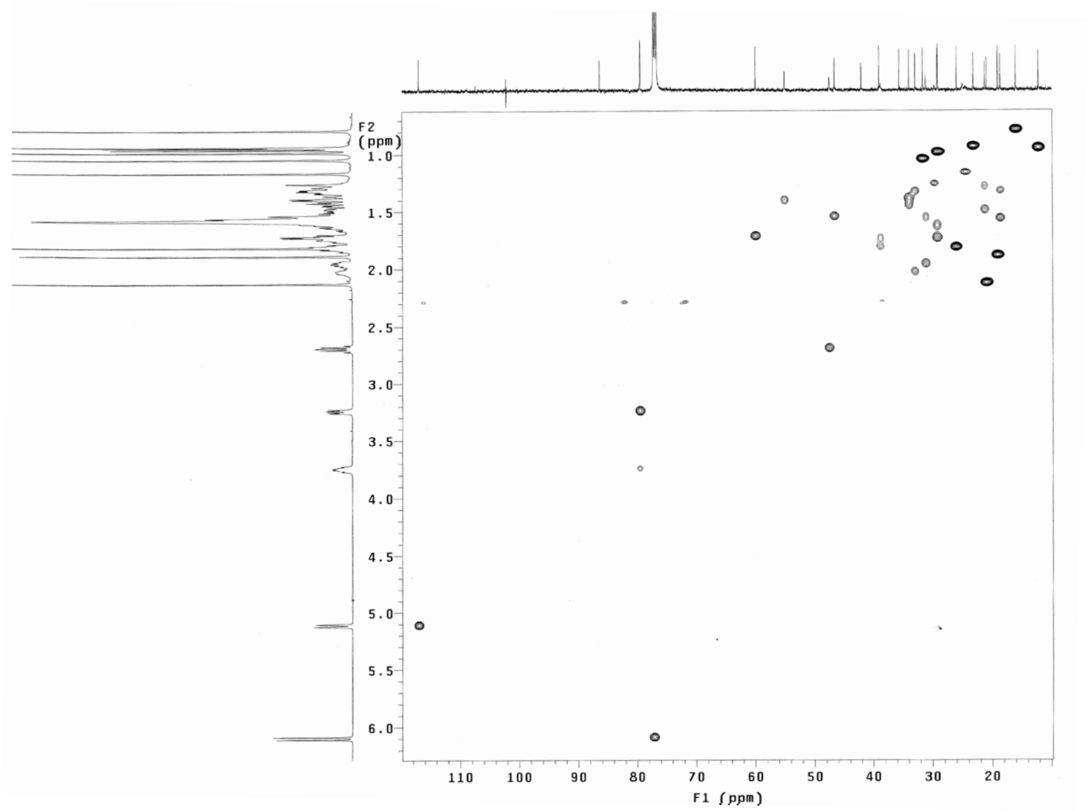

Figure S26. HSQC spectrum of **2**

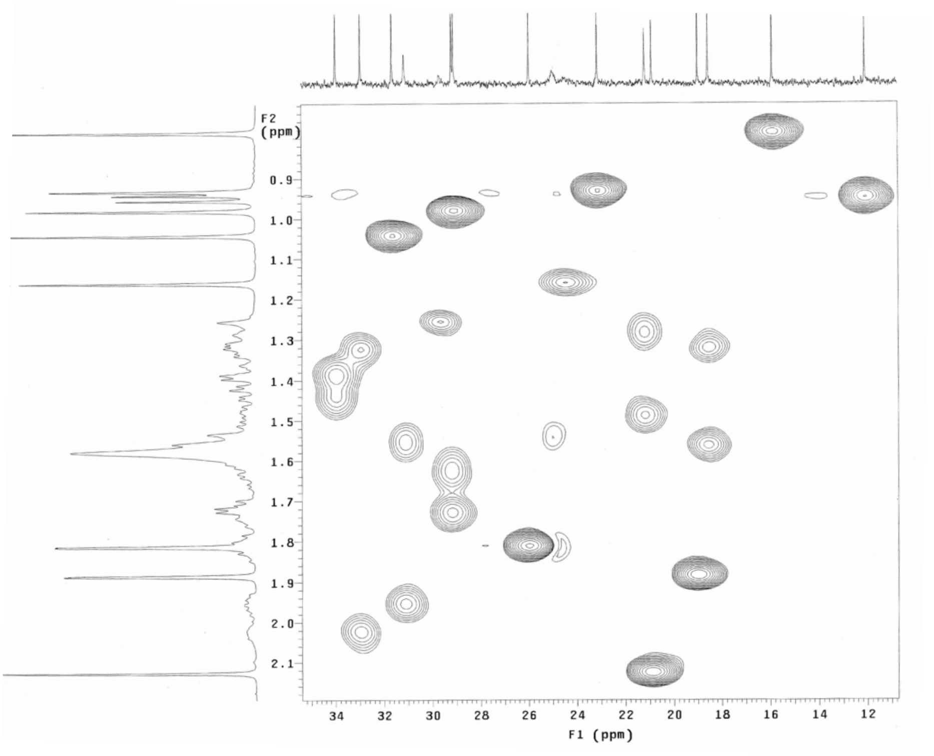

Figure S27. HSQC spectrum of **2** (Partial enlarged view)

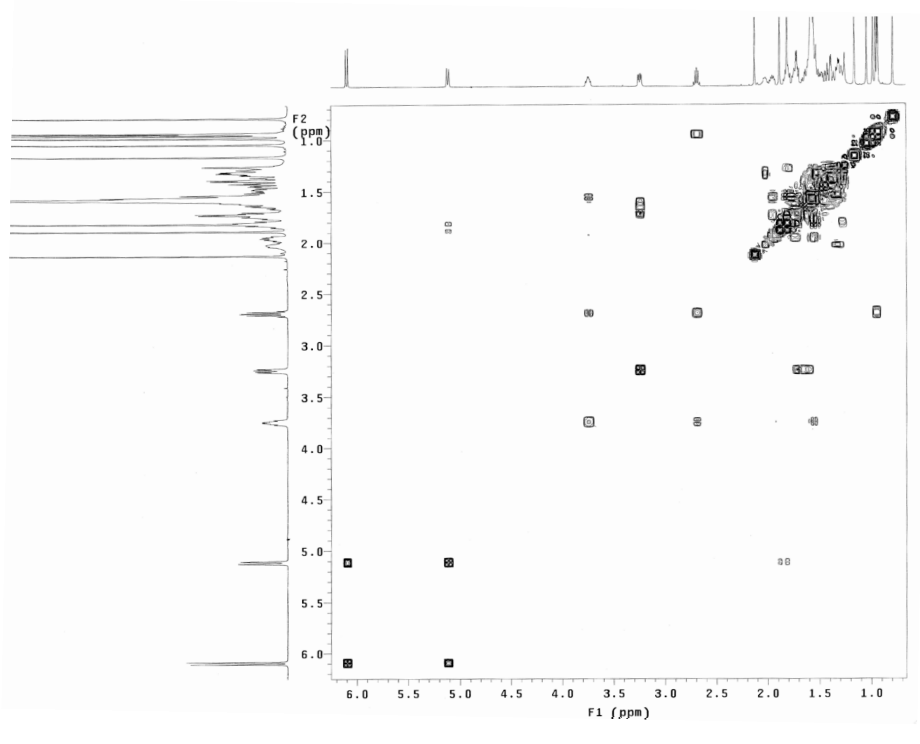

Figure S28. COSY spectrum of **2**

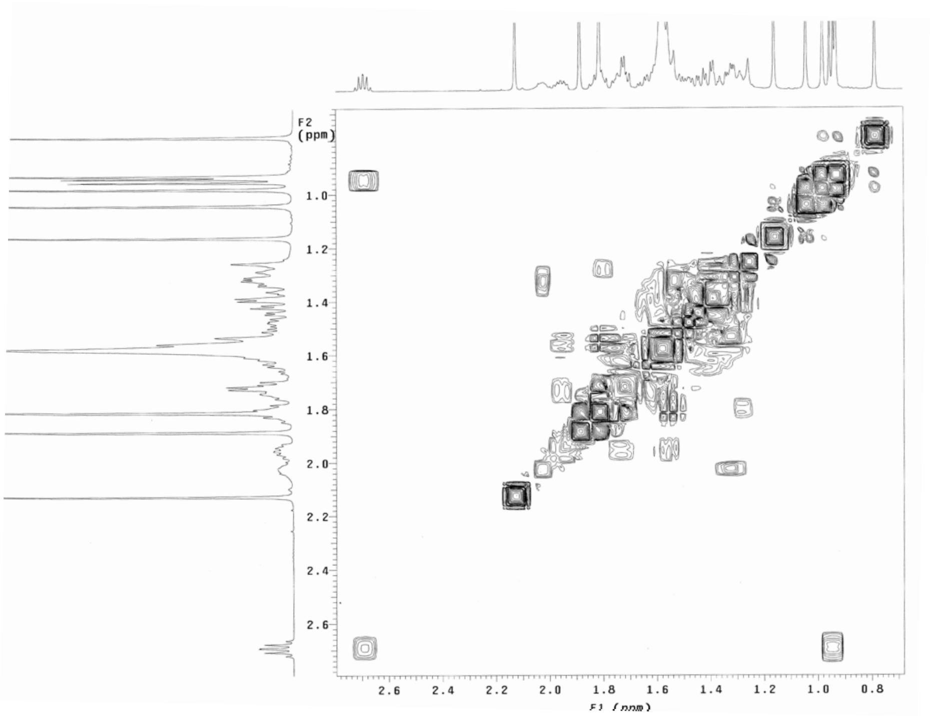

Figure S29. COSY spectrum of **2** (Partial enlarged view)

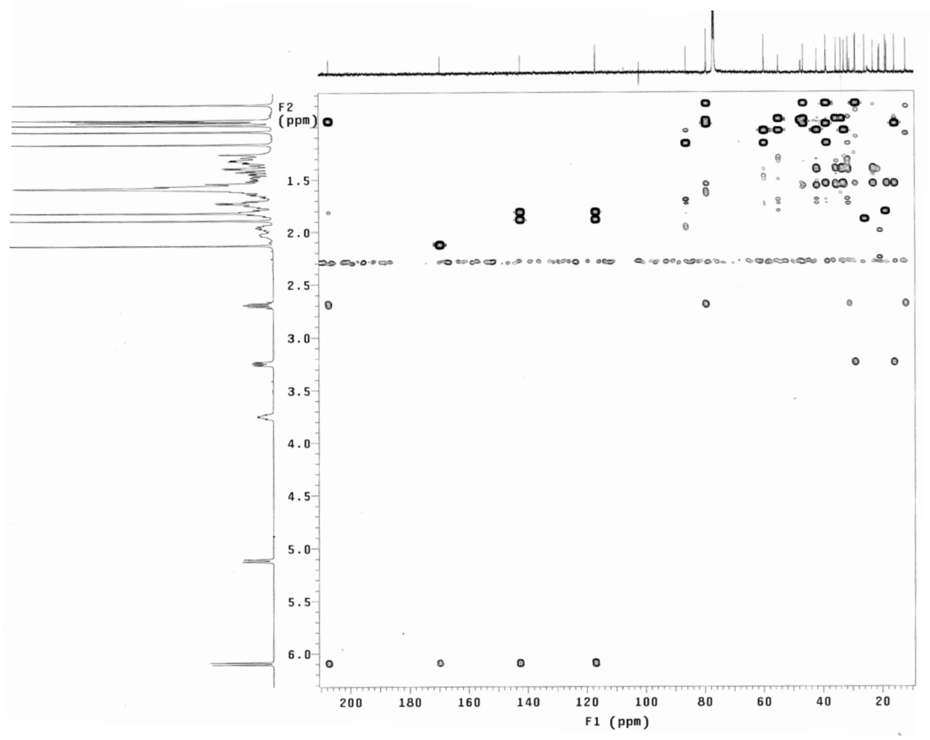

Figure S30. HMBC spectrum of **2**

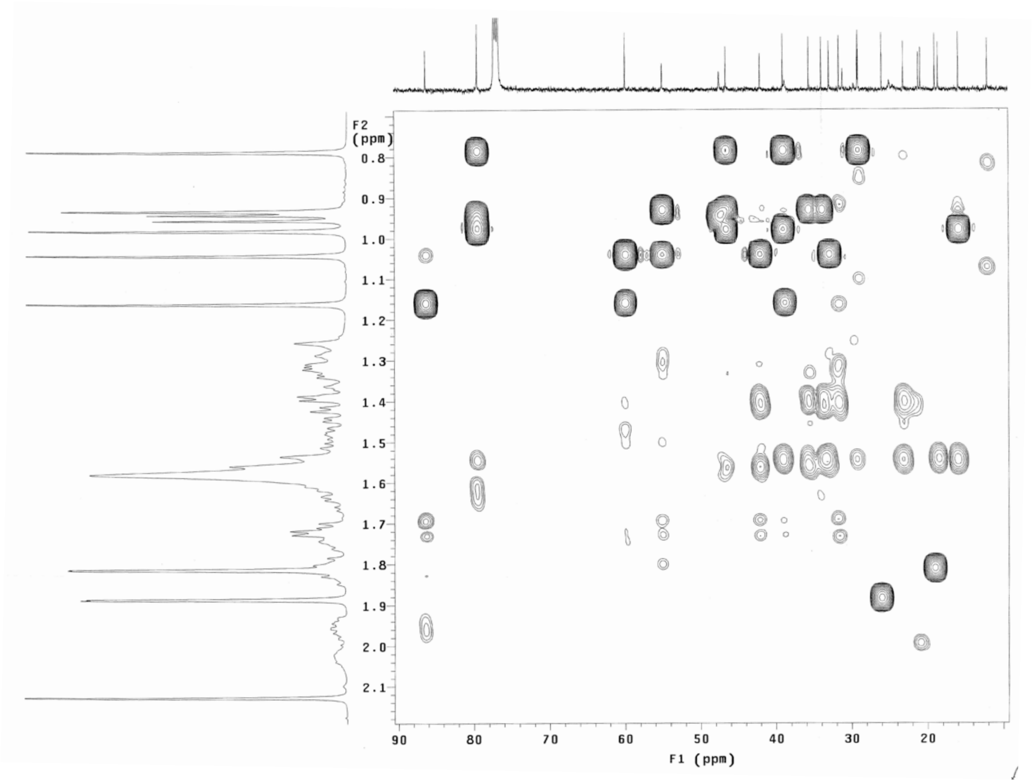

Figure S31. HMBC spectrum of **2** (Partial enlarged view)

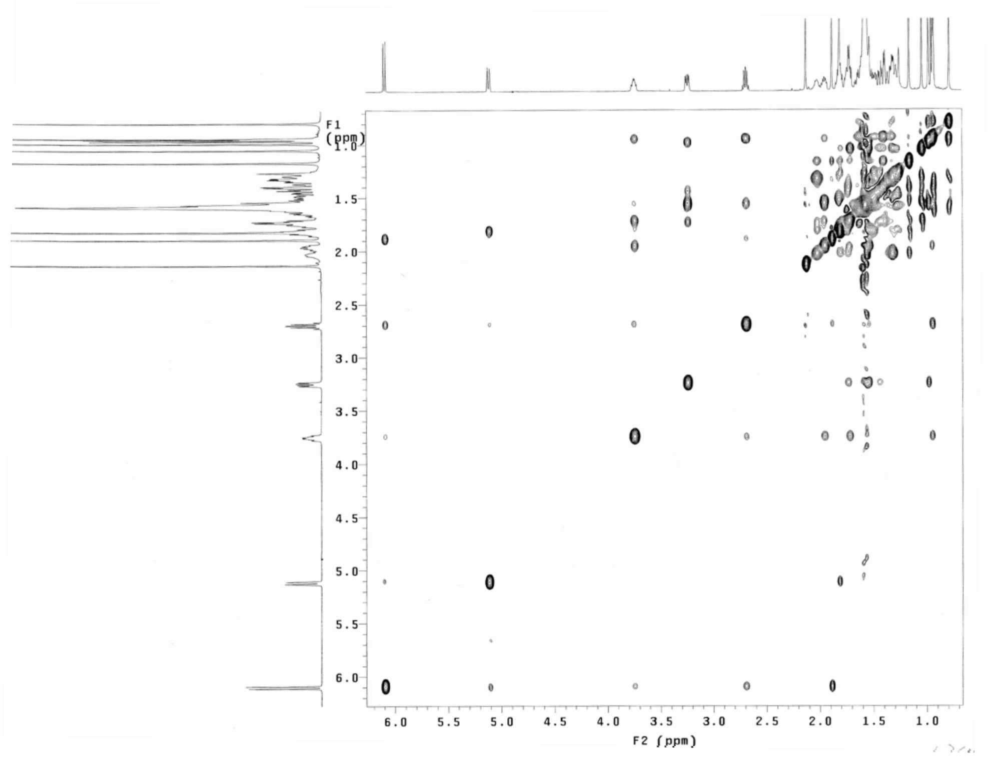

Figure S32. NOESY spectrum of **2**
